# Bcl-2 family inhibitors sensitize human cancer models to therapy

**DOI:** 10.1038/s41419-023-05963-1

**Published:** 2023-07-17

**Authors:** Elisabetta Valentini, Marta Di Martile, Matteo Brignone, Marica Di Caprio, Isabella Manni, Michela Chiappa, Ilaria Sergio, Martina Chiacchiarini, Chiara Bazzichetto, Fabiana Conciatori, Simona D’Aguanno, Carmen D’Angelo, Rino Ragno, Michelangelo Russillo, Gianni Colotti, Francesco Marchesi, Maria Laura Bellone, Fabrizio Dal Piaz, Maria Pia Felli, Giovanna Damia, Donatella Del Bufalo

**Affiliations:** 1 Preclinical Models and New Therapeutic Agents Unit, IRCCS Regina Elena National Cancer Institute, Rome, Italy; 2 SAFU Unit, IRCCS Regina Elena National Cancer Institute, Rome, Italy; 3 Laboratory of Gynecological Preclinical Oncology, Istituto di Ricerche Farmacologiche Mario Negri IRCCS, Milan, Italy; 4 Department of Experimental Medicine, Sapienza University of Rome, Rome, Italy; 5 Oncogenomic and Epigenetic Unit, IRCCS Regina Elena National Cancer Institute, Rome, Italy; 6 Rome Center for Molecular Design, Department of Drug Chemistry and Technology, Sapienza University of Rome, Rome, Italy; 7 Division of Medical Oncology, IRCCS Regina Elena National Cancer Institute, Rome, Italy; 8 Institute of Molecular Biology and Pathology, Italian National Research Council, Rome, Italy; 9 Hematology Unit, IRCCS Regina Elena National Cancer Institute, Rome, Italy; 10 Department of Medicine, Surgery and Dentistry, University of Salerno, Fisciano, Italy

**Keywords:** Cancer therapy, Cancer

## Abstract

BH3 mimetics, targeting the Bcl-2 family anti-apoptotic proteins, represent a promising therapeutic opportunity in cancers. ABT-199, the first specific Bcl-2 inhibitor, was approved by FDA for the treatment of several hematological malignancies. We have recently discovered IS21, a novel pan BH3 mimetic with preclinical antitumor activity in several tumor types. Here, we evaluated the efficacy of IS21 and other BH3 mimetics, both as single agents and combined with the currently used antineoplastic agents in T-cell acute lymphoblastic leukemia, ovarian cancer, and melanoma. IS21 was found to be active in T-cell acute lymphoblastic leukemia, melanoma, lung, pancreatic, and ovarian cancer cell lines. Bcl-xL and Mcl-1 protein levels predicted IS21 sensitivity in melanoma and ovarian cancer, respectively. Exploring IS21 mechanism of action, we found that IS21 activity depends on the presence of BAX and BAK proteins: complexes between Bcl-2 and Bcl-xL proteins and their main binding partners were reduced after IS21 treatment. In combination experiments, BH3 mimetics sensitized leukemia cells to chemotherapy, ovarian cancer cells and melanoma models to PARP and MAPK inhibitors, respectively. We showed that this enhancing effect was related to the potentiation of the apoptotic pathway, both in hematologic and solid tumors. In conclusion, our data suggest the use of inhibitors of anti-apoptotic proteins as a therapeutic strategy to enhance the efficacy of anticancer treatment.

## Introduction

Cancer patients often show intrinsic/innate or acquired resistance to treatments that ultimately result in death [1, 2]; thus, managing these patients represents a challenge, and new therapeutic options are needed. One attractive strategy is the combination of currently available therapies with inhibitors of anti-apoptotic proteins from the Bcl-2 family, which are often expressed at high levels in various types of cancers [3, 4]. Through the interaction with pro-apoptotic proteins, anti-apoptotic proteins allow cells to evade apoptosis, a mechanism frequently defective in cancer affecting the response to therapy [5]. Thus, favoring apoptosis could represent a valuable approach to overcoming resistance.

In 2016, venetoclax/ABT-199, a specific Bcl-2 inhibitor, was approved by the FDA for the treatment of several kinds of leukemia and lymphoma, thus validating the relevance of targeting apoptotic machinery in onco-hematological neoplasms [6]. In addition, the first clinical study analyzing the efficacy of venetoclax in solid tumors evidenced a good activity in metastatic breast cancer [7]. Preclinical results led to the use of combinatorial regimes, including venetoclax or other inhibitors of the Bcl-2 family, in clinical trials under evaluation in cancers of different origins [8, 9].

Experimental findings support the use of anti-apoptotic protein inhibitors in combination therapy both in melanoma and ovarian carcinoma. Inhibition of BRAF and/or MEK/ERK pathways, which together with immunotherapy represent the standard-of-care for melanoma treatment, has been reported to modulate the expression of some members of the Bcl-2 family [10–21]. Analysis of melanoma biopsies after treatment with BRAF inhibitors (BRAFi) further supported these preclinical observations, i.e., demonstrating lower levels of NOXA protein [20] and higher expression of BIM and BID proteins, as well as an inverse correlation between Bcl-2 protein expression and patient outcome [16]. Moreover, Bcl2A1/BFL1 anti-apoptotic protein expression in melanoma patients has been positively correlated with the response to BRAFi [22, 23]. Pro-apoptotic and anti-apoptotic proteins have also been identified as critical mediators of the resistance to BRAFi and MEK inhibitors (MEKi) [15, 17, 24–26]. Similarly, Bcl-2 family members play a role in the apoptosis induced by chemotherapy and in chemoresistance in ovarian cancer [27–29].

Targeting cell death pathways increases the sensitivity of melanoma and ovarian carcinoma models to currently available therapies. Specifically, ABT-737 [30] and ABT-263/navitoclax [31] (BH3 mimetics inhibiting Bcl-2, Bcl-xL, and Bcl-W proteins), TW-37 (Bcl-2, Bcl-xL, and Mcl-1 inhibitor), S63845 and AZD5991 (Mcl-1 inhibitors), have all been found to affect the response of melanoma cells to BRAFi and MEKi [17, 18, 20, 26, 32]. Regarding ovarian cancer, preclinical studies demonstrated that inhibition of Bcl-xL or Mcl-1 restored sensitivity to platinum compounds [28, 33–35] while targeting Mcl-1 and Bcl-2 overcame paclitaxel resistance [36, 37]. Specific inhibition of Bcl-xL has also been reported to improve ovarian cancer’s response to taxane-based therapy and to poly-ADP ribose polymerase inhibitors (PARPi) [29, 38–40]. We have recently identified IS21, a pan-inhibitor of anti-apoptotic proteins with antitumor activity in different histotypes, including melanoma [41]. Here, using in vitro and in vivo approaches, we explored the antitumor efficacy of IS21 and ABT-199 as single agents and their ability to affect the sensitivity of human melanoma, ovarian carcinoma, and T-cell acute lymphoblastic leukemia (T-ALL) models to the standard-of-care agents.

## Materials and methods

### Cell cultures

Human T-ALL (JURKAT, TALL1, LOUCY, MOLT-3), *wild type* (*wt*) melanoma (Sbcl1 ME4405, ME2/17, ME1007), mutated melanoma (M14, A375, LOX IMVI), non-small cell lung cancer (NSCLC) (H1299, A549, HCC827, H460), pancreatic cancer (HPAFII, PANC1, CFPAC1, L3.6) and ovarian cancer (OVCAR 3, OVCAR 5, OVCAR 8, OVCAR 420, OVCAR 432, OVCAR 433) cell lines were maintained in RPMI-1640 complete medium (Euroclone, Milan, IT) containing 10% inactivated fetal bovine serum (FBS) (Hyclone, Thermo Fisher Scientific, Waltham, MA, USA), 1% L-glutamine (Euroclone) and 100 μg/ml penicillin/streptomycin (Euroclone). Cell lines were routinely tested for mycoplasma contamination and authenticated within the last eight months.

### Reagents preparation and treatments

For in vitro experiments, IS21 (ChemSpace, Riga, Latvia), ABT-199, dabrafenib, trametinib, ABT-263, S63845 (Selleckchem, Houston, TX, USA), WEHI-539 (MedChem, Monmouth Junction, USA) and olaparib (Targetmol, Wellesley Hills, MA, USA) were dissolved in DMSO (20 mM, Sigma-Aldrich, St. Louis, Missouri, USA) and further diluted in complete medium. The pan-caspase inhibitor zVAD-fmk (zVAD, Sigma-Aldrich) was dissolved in DMSO. Doxorubicin (50 mg/25 ml, Pfizer, New York, NY, USA) and vincristine (1 mg/ml, Teva Italia, Milan, IT) were serially diluted in complete medium. As control, cells were treated with 0.1%–0.4% DMSO, depending on the highest dose of treatment used for each experiment. DMSO at these concentrations did not affect cell proliferation.

### Cell viability and clonogenic ability

For cytotoxicity experiments, cell lines were plated in 96-well plates and, after 24 h, were treated with different drugs. MTT assay (Sigma-Aldrich) was used for melanoma, pancreatic, and NSCLC cells exposed to single agents, whereas CellTiter-Glo® Luminescent assay (Promega, Southampton, UK) was used for combinatorial regimes in melanoma. MTS (Promega) was used for ovarian cancer cells. For T-ALL, the number of viable cells was counted by a Trypan Blue exclusion assay (Sigma-Aldrich). The count was performed with hand tally counter and hemocytometer (Neubauer cell chamber). Loewe Scores were calculated by SynergyFinder 2.0 software [42]: scores <0, 0≤ scores >5, and ≥5 indicated, respectively, antagonistic, additive, and synergistic effects. GraphPad Prism software (Dotmatics, Bishop’s Stortford, UK) was used to evaluate the concentration of the drug reducing 50% cell viability (IC_50_).

To evaluate the cell colony-forming ability in melanoma cells, after treatment cells were seeded (200 cells) into 60-mm Petri dishes. After 10 days, colonies were stained (2% methylene blue in 95% ethanol) and counted. All cell aggregates with more than 50 cells were considered colonies, while those containing less than or equal to 50 cells were not considered colonies. The surviving fractions were calculated as the ratio of absolute survival of treated sample/survival of the control sample.

For colony assay in OVCAR 5, cells were seeded (250 cells/mL) in 6-well plates and, after 48 h, were treated with olaparib and IS21, and colonies were left to grow for 10 days, then stained (Gram’s Crystal Violet solution, Merck) and quantified (QICAM 32-0030 C camera, QIMAGING, and Colony plus 2.0 program), by setting standardized parameters, considering a cell aggregate containing at least 50 cells (e.g. area min 0.0025, area max 20.0000, background correction, edges exclusion).

### siRNA transfection assay

Pooled siRNA oligonucleotides against BAX (cat L-003308-01-0005), BAK (cat L-003305-00-0005), or scramble (cat D-001810-10-05) target sequences were purchased from DharmaconRNA Technologies (siGENOME SMARTpool, Lafayette, CO, USA). For siRNA transfection, A375 cells were seeded in 6-well plates and, after 24 h, transfected with 20 nM pooled oligonucleotides mixture of BAX or BAK or both BAX/BAK by using JetPrime (Invitrogen, Grand Island, NY, USA) following the manufacturer’s protocol. Gene silencing efficacy by siRNA after 72 h of transfection was assessed by Western blot analysis.

### Immunoprecipitation and western blot analyses

For immunoprecipitation experiments, A375 cells treated for 48 h with vehicle or 20 μM IS21 were washed three times with PBS, harvested, and lysed in RIPA buffer (Santa Cruz Biotechnology, Texas, USA) in the presence of proteases and phosphatases inhibitors. Protein extract was obtained by centrifugation (15 min, 10 000 × *g*, 4 °C) and protein concentrations were measured in duplicate using a BCA (Pierce, Thermo Fisher Scientific) protein assay kit. Protein extracts (1500 μg) were pre-cleared for 2 h at 4 °C by adding 30 μl Dynabeads Protein G (Invitrogen, Thermo Fisher Scientific). The pre-cleared lysates were transferred to a new tube and incubated overnight at 4 °C with Bcl-2 (cat 4223), Bcl-xL (cat 2764), or Mcl-1 (cat 94296) antibodies (1:100, Cell Signaling, Milan, IT) or with 2 μg rabbit IgG (BETHYL Laboratories, Waltham, MA), as control. 60 μl Dynabeads Protein G were washed three times, and then the lysates were added and incubated for 2 h at room temperature. The beads were washed (1 ml PBS, 0.02% Tween) five times, and then the immunoprecipitated proteins were eluted with 30 μl Laemmli sample buffer 2×, without reducing agent, and boiled (10 min, 70 °C). Immunoprecipitated proteins were separated from the beads and 30 μl of water was added to each sample to a final volume of 60 μl. One-third of eluted samples (500 μg of starting materials) for each condition were separated by 12% precast SDS-PAGE gel (Bio-Rad, Hercules, CA, USA).

Western blot analyses were performed as previously described [43, 44]. Immunodetection was performed using antibodies directed to Bcl-2 (cat sc-509), Bcl-xL (cat sc-634) and Mcl-1 (cat sc-12756) (Santa Cruz Biotechnology), BAX (cat 5023 S), BAK (cat 12105 S), BIM (cat 2933 S), NOXA (cat 14766 S) and Beclin-1 (cat 3738 S) (Cell Signaling), PARP (cat 556494, BD biosciences), β-actin (cat A1978, Sigma-Aldrich) and α-tubulin (cat sc-32293, Santa Cruz Biotechnology). Anti-mouse immunoglobulin G-horseradish peroxidase-conjugated antibodies (Invitrogen) were used as secondary antibodies. Images were acquired by Image Lab Software (Bio-Rad), using a ChemiDoc System instrument (Bio-Rad). Densitometric analysis was performed with Image J software version 1.53a (Rasband, W.S., ImageJ, U. S. National Institutes of Health, Bethesda, Maryland, USA, https://imagej.nih.gov/ij/). Values were expressed as fold change of the protein of interest relative to the housekeeping one.

### Cytofluorimetric analysis

To analyze cell cycle distribution, cells were fixed with ice-cold 70% ethanol for 24 h at 4 °C. Next, cells were washed in PBS buffer and stained with 500 μl of PBS containing RNase A (100 μg/ml, Sigma-Aldrich) and Propidium iodide (50 μg/ml) for 30 min in the dark.

For annexin V staining, cells were washed with 1× binding buffer (Invitrogen, Waltham, Massachusetts, USA) and then incubated with APC annexin V (cat. ALX-209-252, Enzo Lifesciences, Farmingdale, NY, USA) following the manufacturer’s instructions.

For active caspase 3 evaluation, cells were fixed and permeabilized with BD Cytofix/Cytoperm (BD Bioscience, San Jose, CA) for 20 min and stained with Alexa Fluor 647 active caspase 3 antibody (cat. 560626, BD Bioscience), following the manufacturer’s instructions. Flow cytometric analyses on melanoma and T-ALL cells were performed using BD Accuri™ C6 and FacsCalibur (BD Biosciences) flow cytometers, respectively. Sample acquisition was performed by setting standardized parameters [blue laser (488 nm), FL2 filter (585/45 nm) for PI staining; red laser (640 nm), FL4 filter (675/25) for annexin V/active caspase 3 staining].

### In vivo experiments

A375 cells were infected with luciferase-encoding lentivirus (pRRLSIN.cPPTLuciferase.WPRE from Addgene, Watertown, Massachusetts, USA) as previously described [45]. 5×10^6^ A375luc cells were subcutaneously injected in the right flank of 6–8-week-old female athymic CD1 nude mice. After 5 days, mice were randomized into different groups (6 mice for each group) and treated for three weeks with dabrafenib (5 mg/kg, oral gavage [o.g.]), trametinib (0.1 mg/kg, o.g.), IS21 (100 mg/kg, intra-peritoneally [i.p.]), ABT-199 (100 mg/kg o.g.) alone or in combinations. IS21 and ABT-199 were dissolved in 10% DMSO, 30% PEG400 (Sigma-Aldrich), 5% Tween80 (Acros Organics, New Jersey, USA), and 55% NaCl (vehicle). Dabrafenib and trametinib were suspended in an aqueous mixture of 0.5% hydroxypropyl methylcellulose (Sigma-Aldrich) and DMSO.

Tumor growth was monitored by bioluminescent imaging once a week. The signal was detected using the IVIS Spectrum CT (Perkin Elmer, Waltham, MA, USA) and analyzed using the Living Image software version 4.7.4. Mice were anesthetized, i.p. injected with 75 mg/kg D-luciferin, and imaged 10 minutes after injection. For ex vivo experiments, after sacrifice, lung, spleen, and liver were explanted, immersed in a solution containing D-luciferin for 10 minutes, and analyzed by bioluminescent imaging. Photon emission was measured in specific regions of interest (ROIs). Data were expressed as photon/second/cm2/steradian. The intensity of bioluminescence was color-coded for imaging purposes. ROIs were also used to evaluate the response evaluation criteria in solid tumors (RECIST) modified for animal experimentation [46] and defined as follows: partial response (PR) for tumor change ≤−50%, stable disease (SD) for −50%< tumor change >35%, progression disease (PD) for tumor change ≥35%.

### Cellular thermal shift assay (CETSA)

To perform CETSA, A375 cells (8 × 10^5^) were seeded into 6-well plates in complete medium. The day after, the cells were treated for 4 h with 20 or 40 µM IS21 or 0.1% (v/v) DMSO, as control. After washing, the cells were collected, suspended in PBS and 6 aliquots were subjected to a 3 min incubation at different temperatures between 30 and 55 °C. Subsequently, the cells were lysed by RIPA buffer and centrifuged. The soluble proteins were separated by a 12% SDS-PAGE and Western blot analysis was carried. Densitometric analyses of the resulting bands were carried out using the ImageJ software as above reported. GAPDH was used as a normalizer. The experiments were performed two times.

### Statistical analysis

In vitro experiments were replicated at least three times, unless otherwise indicated, and the data were expressed as average ± standard deviation (SD) or as average ± standard error of the mean (SEM). Group differences were analyzed with a two-sided paired or unpaired Student’s t-test.

In vivo experiments were repeated twice. Differences between groups, analyzed with *T* test, Wilcox, or Mann–Withney, were considered statistically significant for *p* < 0.05.

Statistical analyses were performed with GraphPad Prism 9 software. Sample sizes were chosen based on preliminary results to ensure a power of 80% and an alpha level of 5%. No data or animals were excluded from the analyses.

## Results

### Bcl-2 family inhibitors reduce in vitro cell viability of human cancer cell lines

We firstly assessed the efficacy of IS21, a compound able to bind Bcl-2, Bcl-xL, and Mcl-1 proteins [41], in tumor histotypes in which Bcl-2 family proteins play a crucial role, such as T-ALL [47], melanoma [48], ovarian cancer [27], pancreatic cancer [49] and NSCLC [50].

**Fig. 1:**
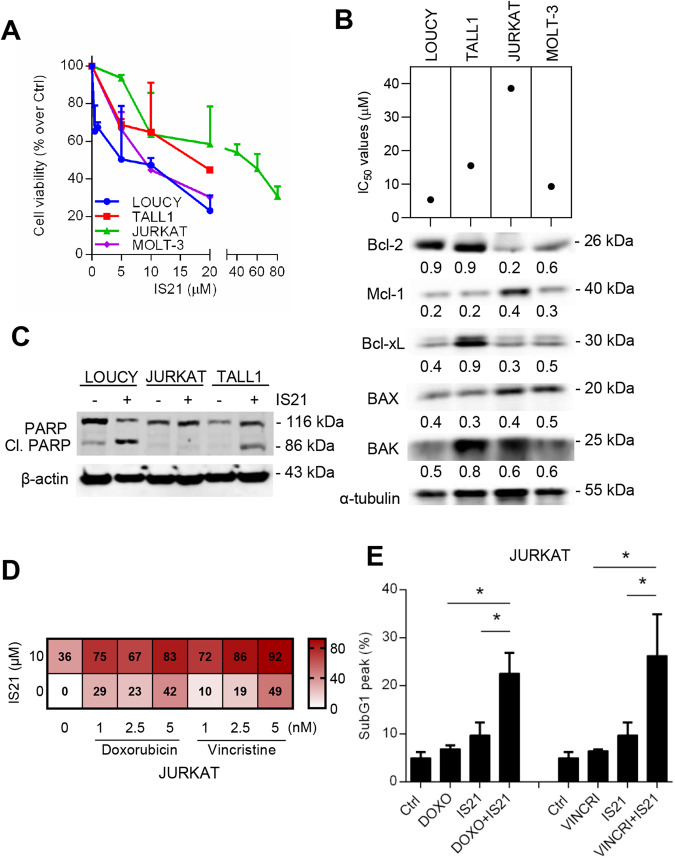
IS21 reduces the viability and potentiates the efficacy of chemotherapy in T-ALL cell lines. **A** Analysis of cell viability of the indicated T-ALL cell lines treated with increasing concentrations of IS21 for 48 h. **B** IC_50_ values of T-ALL cells treated as reported in **A**, and Western blot analysis of basal Bcl-2, Mcl-1, Bcl-xL, BAX, and BAK protein expression levels. The numbers indicate protein quantification by densitometric analysis. **C** Western blot analysis of PARP1 cleavage (Cl. PARP) in the indicated T-ALL cell lines treated with IS21 (20 μM, 48 h). **D** Heat map graph showing cell growth inhibitory effect of JURKAT cells treated with 10 μM IS21 alone or with the indicated concentrations of doxorubicin or vincristine for 48 h. **E** Cytofluorimetric quantification of JURKAT cells in the subG1 peak after treatment with doxorubicin (DOXO, 5 nM), vincristine (VINCRI, 2.5 nM) or IS21 (10 μM), alone or in combination for 48 h. *p*-values were calculated between single and combination treatments, *p < 0.05. **B**, **C** Reported Western blot images are representative of two independent experiments with similar results. β-actin and α-tubulin are shown as loading and transferring control, molecular weights are expressed in kilodalton (kDa). The results are reported as **A** “viability of treated cells/viability of control cells (Ctrl)” × 100, and **D** inhibition of cell proliferation of treated cells/inhibition of control cells × 100, and as the mean ± SD of three independent experiments.

IS21 reduced the viability of four T-ALL cell lines differently, with IC_50_ values at 48 h ranging from 5.4 to 38.43 μM (Fig. 1A). We next evaluated the possible association between IS21 activity and the levels of different anti- and pro-apoptotic Bcl-2 family proteins. IC_50_ values of IS21 failed to correlate significantly with endogenous levels of the analyzed proteins (Fig. 1B). Notably, JURKAT cells, expressing lower levels of Bcl-2 protein, were the most resistant to IS21 (Fig. 1A, B). PARP cleavage, indicative of apoptosis triggering, was induced upon IS21 treatment in LOUCY and TALL1 cells with high levels of Bcl-2 protein. At the same time, it was not detected in the JURKAT cells with low Bcl-2 levels (Fig. 1C).

Accordingly to published data [43], ABT-199, a Bcl-2-specific BH3 mimetic, strongly reduced the viability of TALL1, LOUCY, and MOLT-3 cells, failing to significantly suppress JURKAT cell viability (Supplementary Fig. 1A). We next explored the cytotoxic effect of IS21 in combination with doxorubicin and vincristine, two of the main chemotherapy drugs used in the first-line treatment of T-ALL [51]. We focused on the JURKAT cell line, the most resistant cell line to IS21 (Fig. 1A, B), demonstrating that IS21 was able to synergistically potentiate the efficacy of both drugs (Fig. 1D), with Loewe scores of 35.9 and 39.6 for doxorubicin and vincristine respectively. Importantly, IS21 + doxorubicin or vincristine treatments significantly increased the percentage of apoptotic cells compared to single agents (Fig. 1E).

**Fig. 2:**
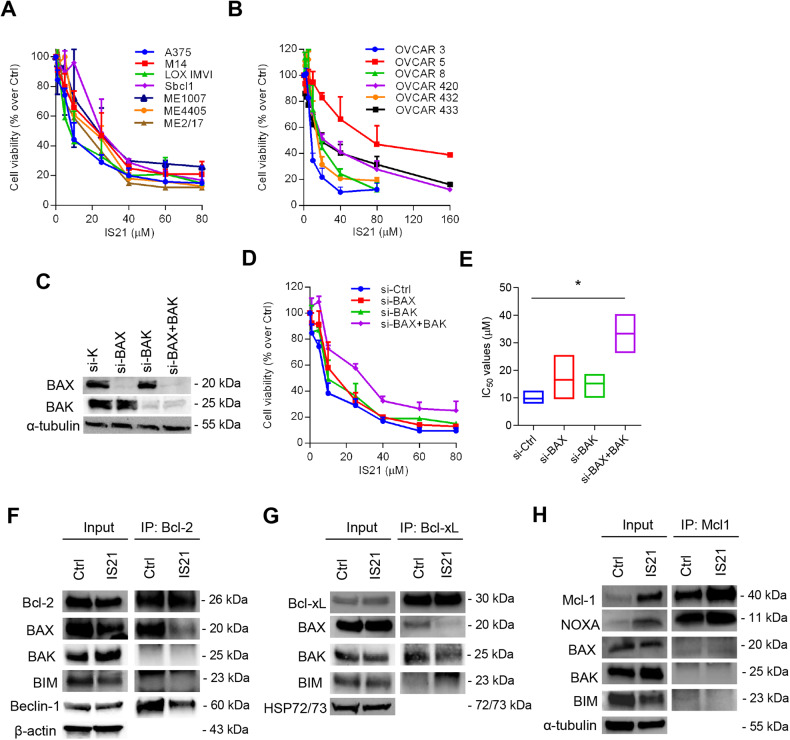
IS21 reduces the viability of solid tumor cell lines and alters the interactions between Bcl-2 and Bcl-xL with their targets. Analysis of cell viability of **A** melanoma and **B** ovarian cancer cell lines treated with increasing concentrations of IS21 for respectively 72 h and 5 days. Results are reported as “viability of treated cells/viability of control cells (Ctrl)” × 100, and as mean ± SD of three independent experiments. **C** Western blot analysis of BAX and BAK protein levels in A375 melanoma cell lines control (si-K), silenced for BAX (si-BAX), BAK (si-BAK), or both proteins (si-BAX + BAK). The same filter was used first for BAK and then for BAX. Analysis of **D** cell viability and **E** relative IC_50_ values of A375 melanoma cells silenced as reported in **C** and treated as reported in **A**. *p*-value was calculated between control and cells silenced for BAX, BAK or both, *p ≤ 0.05. **F**–**H** Western blot analysis of Beclin-1, anti-apoptotic (Bcl-2, Bcl-xL, Mcl-1), and pro-apoptotic (BAX, BAK, BIM, NOXA) proteins after immunoprecipitation of A375 melanoma cells with **F** Bcl-2, **G** Bcl-xL, or **H** Mcl-1 antibodies after treatment with 20 μM IS21 for 48 h. Control cells (Ctrl). **C**, **F**–**H** Reported western blot images are representative of two independent experiments with similar results. β-actin, α-tubulin, and Hsp72/73 are shown as loading and transferring control, molecular weights are expressed in kilodalton (kDa).

Moving on to solid tumors, a heterogeneous response was observed, with IS21 IC_50_ values ranging from 9.1 μM to 27.1 μM for melanoma, from 10.1 μM to 81.7 μM for ovarian carcinoma, from 7.7 μM to 35.9 μM for NSCLC, and from 21.4 μM to 65 μM for pancreatic carcinoma (Fig. 2A, B, Supplementary Fig. 1B, C).

Focusing on melanoma and ovarian carcinoma, we assessed the possible associations between IS21 activity and protein levels of a panel of anti- (Bcl-2, Mcl-1, Bcl-xL) and pro- (BAX, BAK, BIM, NOXA) apoptotic proteins. Importantly, we found a significant negative correlation between IS21 IC_50_ values and Bcl-xL protein levels in melanoma cells (*R* = −0.91, *p* = 0.004) (Supplementary Fig. 1D, E) and those of Mcl-1 in ovarian cancer cells (*R* = −0.93, *p* = 0.006) (Supplementary Fig. 1F, G).

To examine whether IS21 activity was dependent on the presence of the pro-apoptotic proteins BAX and BAK, we tested the effect of IS21 in the proliferation of A375 melanoma cells silenced for BAX, BAK, or both proteins (Fig. 2C). Interestingly, BAX + BAK double silenced cells were significantly more resistant to IS21 compared with control cells, being IC_50_ values 10.9 ± 2.4 μM and 33.3 ± 10 μM for control transfected and BAX + BAK silenced cells, respectively (Fig. 2D, E). On the contrary, BAX or BAK single silencing was not sufficient to significantly increase the IS21 IC_50_ value (Fig. 2D, E).

To better understand the IS21 mechanism of action, we next explored how IS21 treatment altered the interactions between its targets (Bcl-2, Bcl-xL, and Mcl-1) and their main binding partners (BAX, BAK, BIM, NOXA, Beclin-1) in A375 melanoma cells (Fig. 2F–H). Among the protein analyzed, we demonstrated that in control cells, Bcl-2 forms complexes with BAX or Beclin-1 (Fig. 2F), Bcl-xL with BAX or BAK (Fig. 2G), and Mcl-1 with NOXA (Fig. 2H). The treatment with IS21 reduced complexes between Bcl-2 and BAX or Beclin-1 (Fig. 2F), between Bcl-xL and BAX or BAK (Fig. 2G), but not those of NOXA with Mcl-1 (Fig. 2H). In agreement, docking experiments performed with Bcl-2, Bcl-xL, and Mcl-1 complexed with the conserved BH3 α-helix revealed that IS21 binds with an average lower energy into Bcl-2 and Bcl-xL than into Mcl-1 (Supplementary Table 1).

We also evaluated the capability of IS21 to interact actually with the three anti-apoptotic proteins in the cell environment by CETSA [52]. To this purpose, A375 cells were treated for 4 h with IS21 (20 and 40 μM), exposed for 3 min to different temperatures (30–55 °C), and then subjected to non-denaturing lysis to measure the residual amount of soluble Bcl-2, Bcl-xL, and Mcl-1 proteins by Western blot analysis. The results obtained, showed an evident stabilization of Bcl-2 protein at 45 °C and, to a lesser extent, at 50 °C in the samples incubated with 20 or 40 μM IS21, thus confirming the occurrence of an effective Bcl-2/IS21 interaction in the A375 cells (Supplementary Fig. 2A). Conversely, we did not observe any effect of the IS21 treatment on Bcl-xL and Mcl-1 proteins thermal stability (Supplementary Fig. 2B, C).

**Fig. 3:**
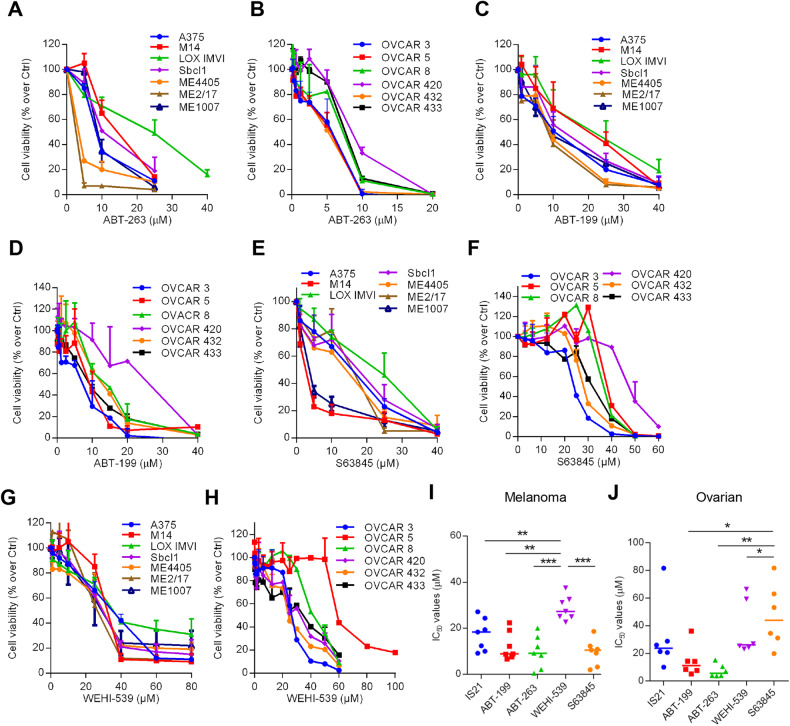
BH3 mimetics differentially affect cell viability of melanoma and ovarian cancer cell lines. Cell growth inhibitory effect of (**A**, **C**, **E, G**) melanoma and (**B**, **D**, **F**, **H**) ovarian cancer cells treated with the indicated concentrations of **A**, **B** ABT-263, **C**, **D** ABT-199, **E**, **F** S63845, and **G**, **H** WEHI-539 for 72 h. IC_50_ values of **I** melanoma and **J** ovarian cancer cell lines treated as described in **A**, **C**, **E**, **G** and **B**, **D**, **F**, **H**, respectively. Results represent the mean ± SD of three independent experiments. **I**, **J** *p*-values were calculated between cells treated with each compound to all the others, *p < 0.05, **p < 0.01, and ***p < 0.001.

We next tested the effect of a panel of pan or specific Bcl-2 family inhibitors on the cell viability of melanoma and ovarian cancer cells. When melanoma (Fig. 3A) and ovarian cancer (Fig. 3B) cells were exposed to increasing concentrations of the pan-inhibitor, ABT-263, a strong reduction of cell proliferation was observed, even if higher heterogeneity was observed in melanoma (IC_50_ values from 2.5 μM to 20 μM) than in ovarian cancer (IC_50_ values from 3.7 μM to 8.8 μM) cells. ABT-199, S63485 (an Mcl-1 specific inhibitor), and WEHI-539 (a Bcl-xL specific inhibitor) caused a dose-dependent reduction of cell viability in melanoma (6.6 μM ≤ IC_50_ ≥ 22.4 μM for ABT-199; 3.2 μM ≤ IC_50_ > 18.6 μM for S63845 and 22.6 μM ≤ IC_50_ ≥ 37.5 μM for WEHI-539) (Fig. 3C, E, G, I) and ovarian carcinoma (5 μM ≤ IC_50_ ≥ 36 μM for ABT-199, 19.8 μM ≤ IC_50_ ≥ 81.7 μM for S63845 and 23.2 μM ≤ IC_50_ ≥ 59.3 μM for WEHI-539) (Fig. 3D, F, H, J), even with a different extent. In fact, melanoma (Fig. 3I) and ovarian cancer (Fig. 3J) cells were more resistant, respectively, to WEHI-539 and S63845 than to the other inhibitors tested.

### BH3 mimetics synergistically potentiate the effect of PARP inhibition in ovarian cancer

**Fig. 4:**
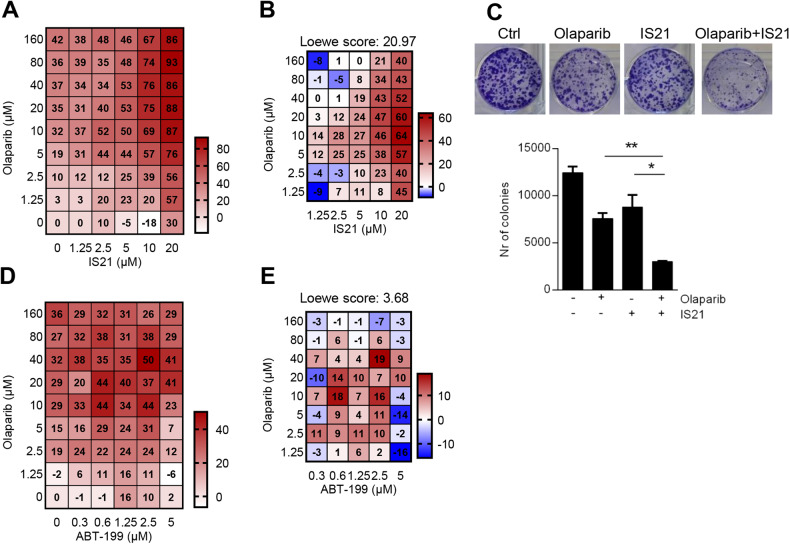
BH3 mimetics sensitize OVCAR 5 cells to olaparib. Heat map graphs showing **A** cell growth inhibitory effect and **B** Loewe scores of cells treated with the indicated concentrations of IS21 and olaparib alone or in combination for 5 days. **C** Representative images (upper panel) and relative quantification (lower panel) of colony assay of cells treated for 10 days with olaparib (1.25 μM) and IS21 (10 μM) alone or in combination. Results represent the mean ± SD of three independent experiments. *p*-values were calculated between cells treated in combination and those treated with single agents, *p < 0.05, **p < 0.01. Heat map graphs showing **D** growth inhibitory effect and **E** Loewe scores of cells treated with the indicated concentrations of ABT-199 and olaparib alone or in combination for 5 days. **A**, **D** The results are reported as inhibition of treated cells/inhibition of control cells × 100, and as mean of three independent experiments. **A**, **B**, **D**, **E** The numbers inside the squares indicate values in every experimental point.

We next investigated the effect of Bcl-2 family inhibitors on the sensitivity of ovarian cancer cells to olaparib, a PARP inhibitor approved for the maintenance setting in ovarian cancer [53], that has been reported to alter the expression of Bcl-2 family proteins in ovarian cancer cell lines [38]. We focused on OVCAR 5, the most resistant cell line to IS21 (Figs. 2B, 3J) and among the most olaparib-resistant ovarian cancer cells (Supplementary Fig. 3A). The treatment with olaparib increased the expression of Bcl-2 protein, while it did not affect the expression of other Bcl-2 family proteins (Supplementary Fig. 3B). Exposure to increasing concentrations of olaparib+IS21 combination elicited a stronger growth inhibitory effect than that obtained with single agents (Fig. 4A). Notably, the two compounds were synergistic in almost all the experimental points (Loewe score ≥5), with an overall Loewe score = 20.97 (Fig. 4B). Furthermore, the combined treatment significantly reduced the clonogenic ability when compared to single agents (Fig. 4C).

Similarly, a synergism (Loewe score = 32.76) was obtained by treating cells with increasing concentrations of olaparib+ABT-263 combination (Supplementary Fig. 3C, D), while an additive effect (Loewe score = 3.68) was observed after ABT-199+olaparib combination (Fig. 4D, E).

### IS21 and ABT-199 synergistically potentiate the effect of MEK inhibition in BRAF*wt* melanoma cells

Since BRAF mutation drives the therapeutic decision in metastatic melanoma patients [54], we compared the IS21 and ABT-199 IC_50_ values between BRAF*wt* and BRAF mutated melanoma cells (Supplementary Fig. 4A), evidencing a not significantly difference between the two groups.

**Fig. 5:**
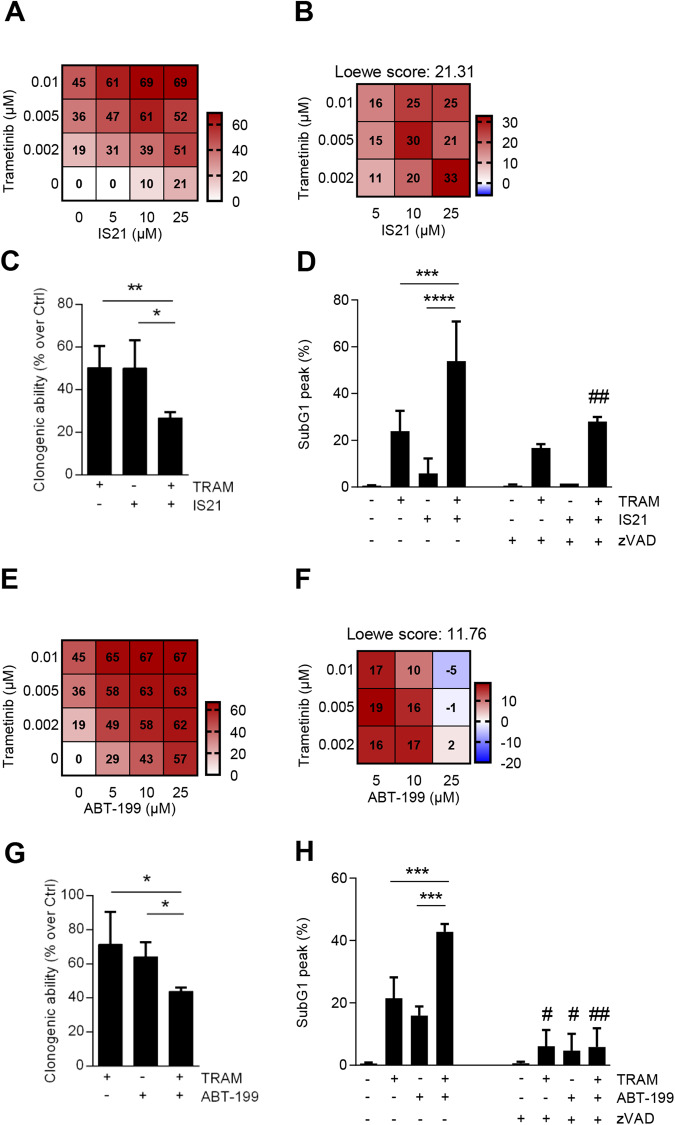
IS21 and ABT-199 potentiate the effect of trametinib in BRAF *wild type* Sbcl1 melanoma cells. Heat map graphs showing **A** cell growth inhibitory effect and **B** Loewe scores of cells treated with the indicated concentrations of IS21 and trametinib alone or in combination for 48 h. **C** Quantification of colony assay of cells treated for 48 h with trametinib (TRAM, 0.01 μM) and IS21 (25 μM) alone or in combination and then plated for 10 days, before colony evaluation. **D** Cytofluorimetric quantification of cells in the subG1 peak after treatment with trametinib (0.01 μM) or IS21 (25 μM), alone or in combination for 48 h, in the presence or absence of zVAD (50 μM). Heat map graphs showing **E** cell growth inhibitory effect and **F** Loewe scores of cells treated with the indicated concentrations of ABT-199 and trametinib alone or in combination for 48 h. **G** Quantification of colony assay of cells treated for 48 h with trametinib (0.005 μM) or ABT-199 (10 μM) alone or in combination and then plated in 60 mm plate (200 cells) for 10 days. **H** Cytofluorimetric quantification of cells in the subG1 peak after treatment with trametinib (0.005 μM), or ABT-199 (10 μM) alone or in combination for 48 h, in the absence or presence of zVAD (50 μM). **A**, **E** The results are reported as inhibition of treated cells/inhibition of control cells × 100 and represented the mean of three independent experiments. **A**, **B**, **E**, **F** The numbers inside the squares indicate values in every experimental point. **C**, **D**, **G**, **H** Data are reported as mean ± SD of three independent experiments. *p*-values were calculated between single and combination treatments, *p < 0.05; **p < 0.01; ***p < 0.001; ****p < 0.0001, and between cells with or without zVAD, ^#^*p* < 0.05 and ^##^*p* < 0.01.

Next, we analyzed the effect of IS21 and ABT-199 combined with the MEKi, trametinib, in Sbcl1 BRAF*wt* melanoma, whose therapeutic options are much less than BRAF mutated ones [55]. Trametinib treatment in Sbcl1 strongly increased the levels of BIM protein, while it did not affect the expression of other Bcl-2 family proteins (Supplementary Fig. 4B). The simultaneous exposure to IS21 and trametinib induced a more potent inhibition of cell proliferation compared to single treatments: a synergistic effect of the two drugs in every experimental point tested was observed, with an overall Loewe score of 21.31 (Fig. 5A, B). Furthermore, the combined treatment significantly reduced the clonogenic ability compared with single agents (Fig. 5C). Analysis of cell cycle distribution evidenced an increased percentage of cells in the subG1 peak, indicative of cell death, after combined treatment (Fig. 5D, Supplementary Fig. 5A), and addition of zVAD to the combination, strongly decreased the percentage of cells in the subG1 peak (Fig. 5D).

The combined treatment trametinib+ABT-199 produced a synergistic effect (Loewe score = 11.76) on growth inhibition (Fig. 5E) when Sbcl1 cells were treated with doses of ABT-199 lower than 25 µM (Fig. 5F). A significant reduction of clonogenic ability (Fig. 5G) paralleled by a significant increase of cells in the subG1 peak (Fig. 5H, Supplementary Fig. 5A), was observed after combined treatment compared with single agents. The addition of zVAD significantly reduced the percentage of cells in the subG1 peak (Fig. 5H).

### IS21 and ABT-199 sensitize BRAF mutant melanoma cells to MAPKi in vitro

**Fig. 6:**
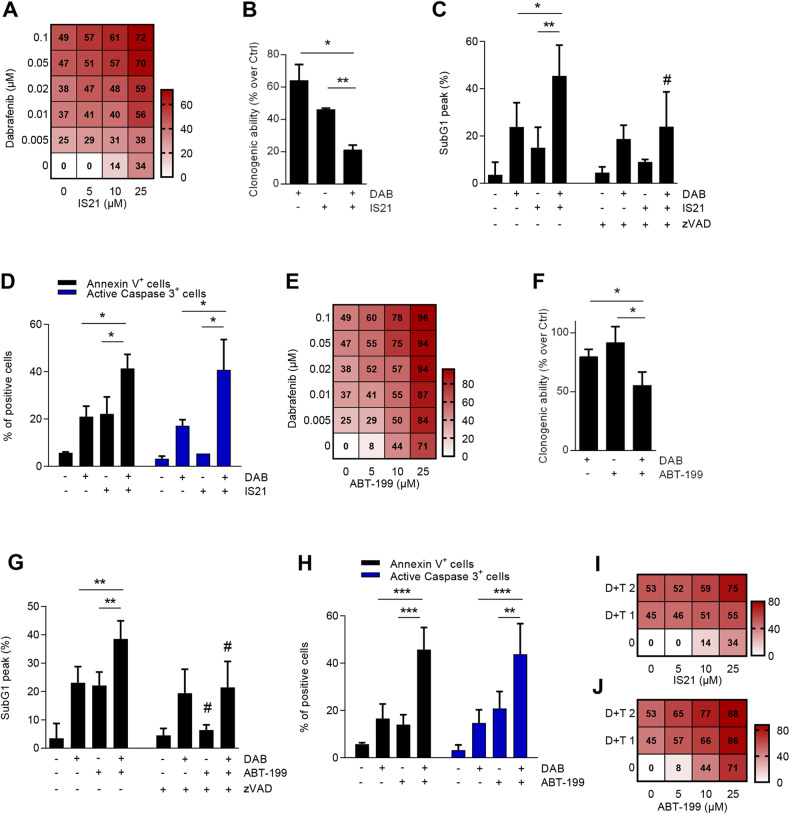
IS21 and ABT-199 cooperate with dabrafenib to reduce the viability of A375 melanoma cells. **A** Heat map graph showing the cell growth inhibitory effect of cells treated with the indicated concentrations of IS21 and dabrafenib alone or in combination for 48 h. **B** Quantification of colony assay of cells treated for 48 h with dabrafenib (DAB, 0.1 μM) and IS21 (25 μM) alone or in combination and then plated in 60 mm plate (200 cells) for 10 days, before colony evaluation. Cytofluorimetric quantification of **C** cells in the subG1 peak and **D** annexin V positive cells and activated caspase 3 in cells treated as reported in **B**, in the absence or presence of zVAD (50 μM). **E** Heat map graph showing cell growth inhibitory effect of cells treated with the indicated concentrations of dabrafenib or ABT-199 alone or in combination for 48 h. **F** Quantification of colony assay of cells treated for 48 h with dabrafenib (0.05 μM) or ABT-199 (10 μM) alone or in combination and then plated in 60 mm plate (200 cells) for 10 days. Cytofluorimetric quantification of **G** cells in the subG1 peak and **H** annexin V positive cells and activated caspase 3 in cells treated as reported in **F**, in the absence or presence of zVAD (50 μM). **I**, **J** Heat map graph showing the cell growth inhibitory effect of A375 cells treated with the indicated concentrations of **I** IS21, **J** ABT-199, dabrafenib+trametinib (D + T 1, dabrafenib 0.01 μM+trametinib 0.001 μM; D + T 2, dabrafenib 0.05 μM+trametinib 0.005 μM); alone or in combination for 48 h. **A**, **E**, **I**, **J** The results are reported as inhibition of treated cells/inhibition of control cells × 100 and represented the mean of three independent experiments. The numbers inside the squares indicate values in every experimental point. **B**–**D**, **F**–**H** Data are reported as mean ± SD of three independent experiments. *p*-values were calculated between single and combined treatments, *p < 0.05; **p < 0.01; ***p < 0.001, and between cells with or without zVAD, ^#^*p* < 0.05.

BRAFi, in combination with MEKi, represents the standard-of-care for advanced BRAF-mutated melanoma [54]. In agreement with previously published papers demonstrating BIM upregulation after BRAFi treatment [18, 56], BRAFi altered the expression of Bcl-2 family proteins in A375 melanoma cells, as demonstrated by the reduction of Bcl-2 and the increase of BIM proteins (Supplementary Fig. 3B). Importantly, the simultaneous treatment of IS21 and dabrafenib, a BRAFi, inhibited the proliferation of both A375 (Fig. 6A, Supplementary Fig. 4C) and M14 (Supplementary Fig. 4D, E) cells with a synergistic interaction (Loewe score = 12.6 for A375 and 16.2 for M14). The combination treatment also reduced the clonogenic ability of A375 cells compared with single compounds (Fig. 6B). A significant increase of cells in the subG1 peak was also observed: 45% after combined treatment compared with single treatment with dabrafenib (23%) or IS21 (15%) in A375 cells (Fig. 6C, Supplementary Fig. 5B) and of 30% in the combination compared with <10% of single agents in M14 cells (Supplementary Fig. 4F). The addition of zVAD significantly reduced the percentage of cells in the SubG1 peak in A375 cells treated with the combination (Fig. 6C). Enhanced apoptosis after the combined treatment was also confirmed by significant increase of annexin V and active caspase 3 positive cells when compared to single agents (Fig. 6D, Supplementary Fig. 5C, D).

Similarly, the combined treatment of A375 and M14 melanoma cells with ABT-199 and dabrafenib induced a synergistic effect in terms of cell viability loss when compared to dabrafenib or ABT-199 alone (Fig. 6E, Supplementary Fig. 4G–I), paralleled by a reduction of clonogenic ability (Fig. 6F), enhancement of cells in subG1 peak (Fig. 6G, Supplementary Fig. 4F, Supplementary Fig. 5B) and apoptotic cells (Fig. 6H, Supplementary Fig. 5C, D).

Notably, IS21 synergistically potentiated the effect of dabrafenib+trametinib combination in A375 (Loewe score = 9.7) and M14 (Loewe score = 8.6) cells (Fig. 6I, Supplementary Fig. 4L–N). Similar results were obtained after ABT-199+dabrafenib+trametinib triple combination both in A375 (Loewe score = 19.9) and M14 (Loewe score = 12) cells (Fig. 6J, Supplementary Fig. 4L–N).

### IS21 and ABT-199 sensitize BRAF mutant melanoma cells to MAPKi in vivo

**Fig. 7:**
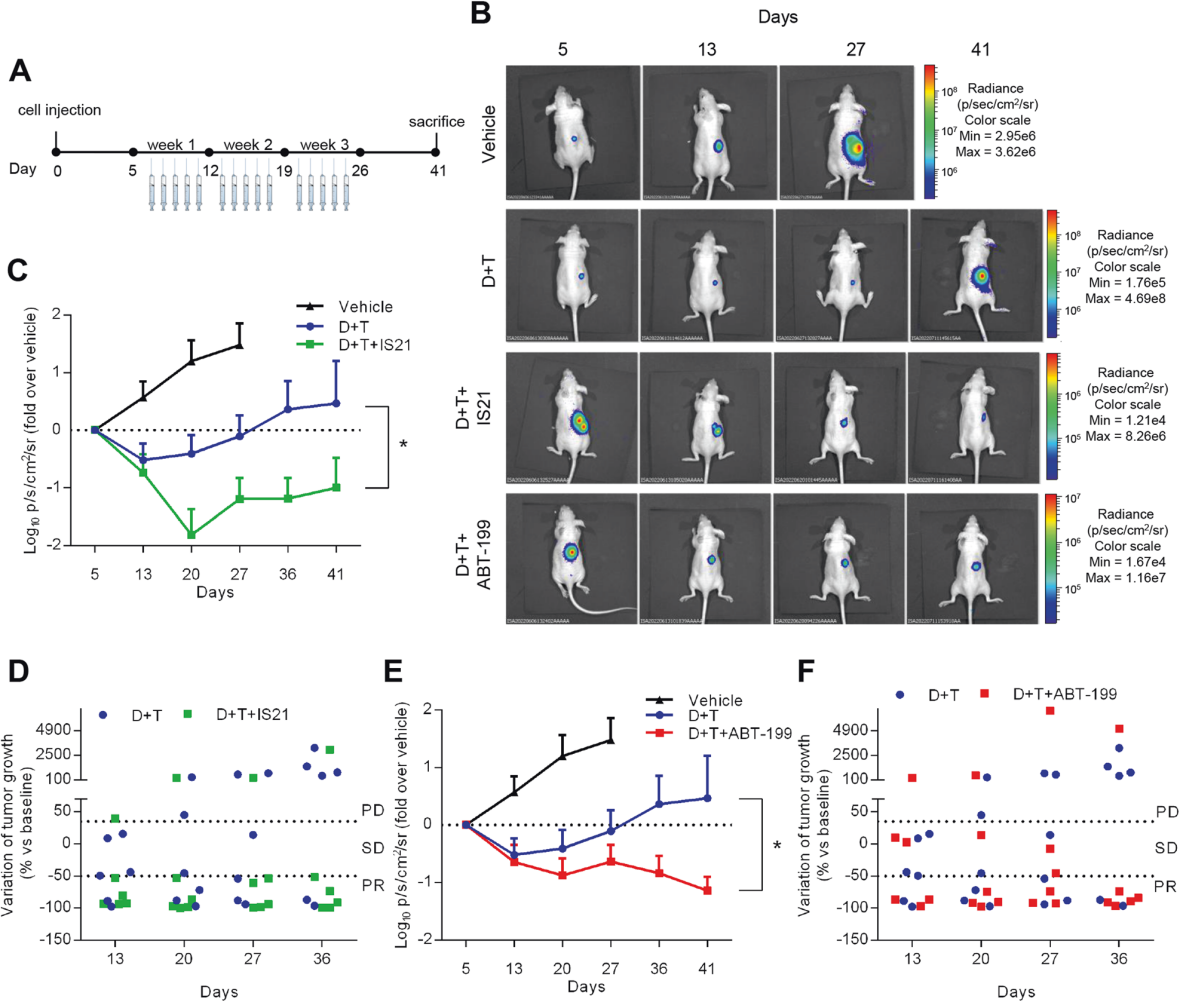
IS21 and ABT-199 potentiate the effect of dabrafenib+trametinib in A375-derived xenografts. **A** Schematic timeline of in vivo experiments. **B** Representative images of in vivo tumor growth analysis in nude mice injected with A375luc cells and treated with vehicle or with dabrafenib+trametinib (D + T) or D + T + IS21 or D + T + ABT-199 for three weeks. **C**, **E** Analysis of tumor growth after A375luc injection and treatment as reported in **B**. **D**, **F** Therapeutic response of single mice treated as reported in **B** assessed by using RECIST-like criteria; progression disease (PD ≥ 35% increase from baseline), partial response (PR ≥ −50% increase from baseline), stable disease (SD, intermediate changes). Experiments were repeated twice, *p < 0.05.

IS21 and ABT-199, administered as single agents, were previously reported to reduce in vivo tumor growth of melanoma models [41]. To evaluate their effect in combinatorial regimes and to validate our results in vivo, mice carrying A375luc tumors were treated with double (dabrafenib+trametinib) or triple (dabrafenib+trametinib+IS21 or dabrafenib+trametinib+ABT-199) combinations (Fig. 7A). Dabrafenib+trametinib caused an initial tumor shrinkage until day 20 when tumors started to regrowing (Fig. 7B, C, F). Notably, the addition of IS21 significantly reduced the tumor growth compared with dabrafenib+trametinib combination (Fig. 7B, C). Assessing the therapeutic response of single mice by using RECIST-like criteria, we found that 5 out of 6 mice treated with the triple combination reached partial response, compared to 2 out 6 in the dabrafenib+trametinib group (Fig. 7D). Similarly, the triple combination dabrafenib+trametinib+ABT-199 significantly reduced the tumor growth compared with the double combination, achieving a longer disease control until the day of sacrifice (Fig. 7B, E), with 5 out of 6 mice responding to treatments (Fig. 7F). Importantly, the effect of the triple treatments, evaluated by the ex vivo imaging of organs, revealed a reduction, even though not significant, on the ability of A375 to colonize lung, spleen, and liver compared with dabrafenib+trametinib (Supplementary Fig. 6A, B). Of note, all treatments were highly tolerated and no significant changes in body weight (Supplementary Fig. 6C), diet consumption, and postural/behavioral habits were observed.

## Discussion

In the past years, we reported that Bcl-2, Bcl-xL, and Bcl2L10 anti-apoptotic proteins positively affect melanoma progression, thus indicating the relevance of these proteins as therapeutic targets [48, 57–60]. We also identified IS21, a novel compound with high affinity for Bcl-2, Bcl-xL, and Mcl-1 proteins, showing antitumor activity [41].

Here, we demonstrated that IS21 reduced the viability of cell lines derived from different tumor histotypes, confirming the relevance of the Bcl-2 family in T-ALL, melanoma, lung, pancreatic and ovarian carcinomas, and fostering their use in a broad spectrum of cancers.

We also identified Bcl-xL and Mcl-1 protein levels as markers of IS21 sensitivity in melanoma and ovarian cancer cell lines, respectively, and demonstrated that the anti-proliferative effect of IS21 was dependent on BAK and BAX expression.

We also deepened the action of IS21 from a mechanistic point of view. Our analysis of Bcl-2, Bcl-xL, and Mcl-1 immunoprecipitates demonstrated that IS21 induced a re-distribution of some pro-apoptotic proteins, strongly reducing the interaction of either Bcl-2 or Bcl-xL with their main binding partners (BAX and Beclin-1 for Bcl-2; BAX and BAK for Bcl-xL), but not that of Mcl-1. Thus, under the condition of IS21 plus target therapy, BAX and BAK could potentially be activated by BIM, which is increased by target therapy. Our results suggested that BAX and BAK, being the main interactors of Bcl-2 and Bcl-xL, were necessary to the IS21 activity, although their baseline levels did not predict the IS21 sensitivity in T-ALL nor in melanoma or ovarian cancer models.

Moreover, docking experiments indicated that IS21 was able to displace the BH3 helix more effectively from Bcl-2 and Bcl-xL than from Mcl-1. Using CETSA experiments, we confirmed the direct interaction of IS21 with Bcl-2 inside the cancer cell. The lack of interaction between the compound and Bcl-xL or Mcl-1 could depend on a lower affinity of IS21 for these proteins, as already suggested by SPR data [40]. However, it should be emphasized that in many cases the expression level of a protein, the localization, and physiological involvement in intracellular interactions can significantly influence its effective ability to bind a compound inside the cell [61]. In fact, it has been reported that the three proteins are differently expressed and localized in melanoma cells *(*https://www.proteinatlas.org/humanproteome/pathology, accessed on July 13th, 2023) [62] and, therefore, possibly differently reachable from soluble BH3 inhibitors. Next, we demonstrated that a panel of pan or specific Bcl-2 family inhibitors was able to reduce the viability of different cancer cell lines, even if to a different extent, confirming the relevance of the Bcl-2 family also in solid tumors, and fostering their use in a wide spectrum of cancers. In melanoma, a specific Bcl-xL inhibitor was less effective than the others BH3 mimetics. Further analyses are needed to test other Bcl-xL inhibitors to confirm this observation.

In combination experiments, we demonstrated that IS21 sensitized T-ALL cells to the treatment with doxorubicin or vincristine, increasing apoptosis. Interestingly, a phase II clinical trial is recruiting T-ALL and lymphoblastic lymphoma patients to evaluate the efficacy of ABT-199 in combination with chemotherapy, including vincristine and doxorubicin (NCT00501826).

In ovarian cancer cell lines, the pan inhibitors IS21 and ABT-263 synergistically potentiated the effect of olaparib, whereas ABT-199 induced an additive effect. Published data demonstrated that targeting Bcl-xL, but not Bcl-2, sensitized ovarian cancer to PARP inhibition [38, 39]. Higher levels of Bcl-xL protein compared to Bcl-2 and the olaparib-induced increase of Bcl-xL could explain why Bcl-2 specific inhibition was less effective in potentiating PARPi, when compared to pan BH3 mimetics [38]. Moreover, Dutta and colleagues identified a novel interaction between Bcl-2 and PARP1 that interfered with PARP1 activity, and demonstrated that Bcl-2 overexpression phenocopies PARP inhibition in chronic lymphocytic leukemia, breast and lung cancer [63]. We cannot exclude that this interaction can also occur in ovarian cancer, and could play a role in the sensitivity of the combination of PARPi with Bcl-2 specific inhibitors. Based on these preclinical findings, several clinical trials are ongoing to study the efficacy of BH3 mimetics alone or in combination in ovarian cancer, and a recent phase II study demonstrated that navitoclax showed an acceptable tolerance profile but poor activity in women with platinum-resistant/refractory recurrent epithelial ovarian cancer [64]. A phase I study is recruiting women with recurrent high-grade serous carcinoma who have progressed after platinum-containing chemotherapy to evaluate the combination of olaparib and navitoclax (NCT05358639).

Our data also demonstrated that IS21 and ABT-199 reduced melanoma cell viability in a BRAF-independent manner, supporting the fact that both BRAF*wt* and BRAF mutated melanoma cells may benefit from the treatment with Bcl-2 family inhibitors.

In BRAF*wt* melanoma cells, the combination of ABT-199 or IS21 with trametinib was more active than single treatments in terms of reduction of cell proliferation and clonogenic ability, and induction of apoptosis. Apoptosis was rescued when zVAD was added to the combined treatment, confirming that BH3 mimetics increase the sensitivity to trametinib through the apoptotic pathway. The ability of some inhibitors of anti-apoptotic proteins to increase the sensitivity to target therapy in the BRAF*wt*/RAS mutated melanoma subgroup has also been reported. ABT-263 synergizes with MEK1/2i and induces death in melanoma cell lines with BRAF or RAS mutations [18]. These findings open the possibility of treating this specific subgroup of melanoma patients, who have limited access to active therapy, with BH3 mimetics. A phase I/II study is active to test the trametinib and navitoclax combination in KRAS or NRAS mutation-positive patients with metastatic/refractory/unresectable malignant solid neoplasms, including melanoma (NCT02079740).

We also found that ABT-199 and IS21 sensitized BRAF-mutated melanoma cells to dabrafenib and dabrafenib+trametinib treatments in terms of reduction of cell viability and clonogenic ability. Analysis of caspase 3 activation, annexin V positive cells, subG1 peak in the cell cycle distribution, and the use of caspase inhibitors demonstrated increased apoptosis after combined treatments. As we have previously demonstrated that IS21 could induce apoptosis through the alteration of autophagy in different tumors [41], we can hypothesize that the IS21 effect herein reported on cell viability/death may be mediated, at least in part, by the autophagic pathway.

Most notably, both ABT-199 and IS21 strongly potentiated the effect of dabrafenib+trametinib in vivo, by reducing tumor growth and metastatic ability and achieving longer disease control, with no evident signs of toxic effects. These data strongly support the clinical development of BH3 mimetic-based combinations.

The ability of specific or pan inhibitors of anti-apoptotic proteins to increase the efficacy of therapies in melanoma is not a new concept [8], but none of these studies, except the study of Rohrbeck’s group [15], analyzed the effect of ABT-199. In particular, our results were in agreement with those demonstrating the ability of ABT-737 [30] and ABT-263 [31], to potentiate the efficacy of the BRAFi in BRAF-mutated melanoma models [11, 13, 15, 16, 18, 19]. In vitro and in vivo ability of ABT-737 or TW-37 to synergize with MEKi has also been reported in melanoma models carrying BRAF mutation [17, 26]. The inefficacy of ABT-199 to affect the sensitivity of BRAF mutated melanoma cells expressing Bcl-2 protein to BRAFi, demonstrated by Rohrbeck’s group, could be due to different scheduling used [15]. More importantly, ABT-737 and ABT-263 and the Bcl-xL specific inhibitor, A1155, were also found to re-sensitize in vitro/in vivo melanoma models resistant to BRAFi or MEKi [11, 15, 16]. Recently, also Mcl-1 inhibitors showed their efficacy in potentiating the activity of BRAFi in melanoma models [19, 20, 32]. All this evidence supports the relevance of BH3 mimetics for melanoma treatment. To this purpose, a phase I/II study is active to evaluate the efficacy of navitoclax in combination with dabrafenib/trametinib in BRAF mutant melanoma patients (NCT01989585).

As a mechanism underlying the potentiating effect of BH3 mimetics on target therapy observed in ovarian cancer and melanoma, we cannot exclude a possible senolytic effect reported by some Bcl-2 family inhibitors [65, 66]. We can speculate that these inhibitors may potentiate the effect of PARPi and MAPKi through the selective activation of apoptosis in olaparib- and dabrafenib/trametinib-induced senescent cells [67–69], as some preclinical evidence suggest [66, 70–74].

In conclusion, our findings suggest new therapeutic strategies active in poorly responsive melanoma and ovarian carcinoma models, both using agents already approved, i.e., venetoclax, with possible rapid clinical translatability, or agent under development, i.e. IS21. We know that IS21 is effective at micromolar concentrations, but it is a compound that can still be optimized before becoming a drug. In this context, we hypothesized chemical modifications (replacement of benzofurane moiety with other bicyclic heterocycles, insertion of methylene groups) to improve its efficacy.

## Supplementary information

Original Data File

Supplementary file

Checklist

## Data Availability

Data and materials are available upon request to the corresponding author.
