# Supplementary material for: Bcl-2 family inhibitors sensitize human cancer models to therapy
Source: Cell Death Dis. 2023 Jul 17;14(7):441. doi: 10.1038/s41419-023-05963-1 (PMC10352371; doi:10.1038/s41419-023-05963-1)

FIG1B

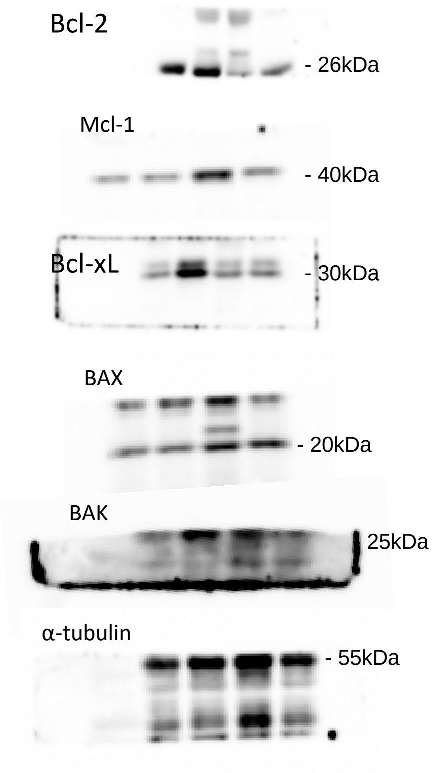

Bcl-2
- 26kDa
Mcl-1
- 40kDa
Bcl-xL
- 30kDa
BAX
- 20kDa
BAK
25kDa
α-tubulin
- 55kDa

FIG1C

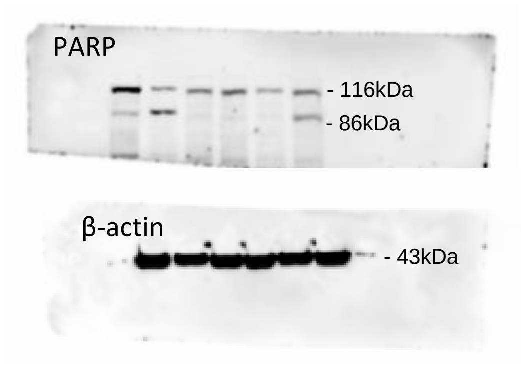

PARP
- 116kDa
- 86kDa
β-actin
- 43kDa

FIG2C

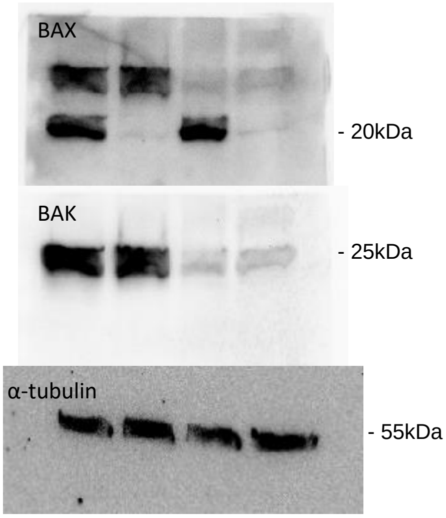
BAX
- 20kDa
BAK
- 25kDa
α-tubulin
- 55kDa

FIG2G

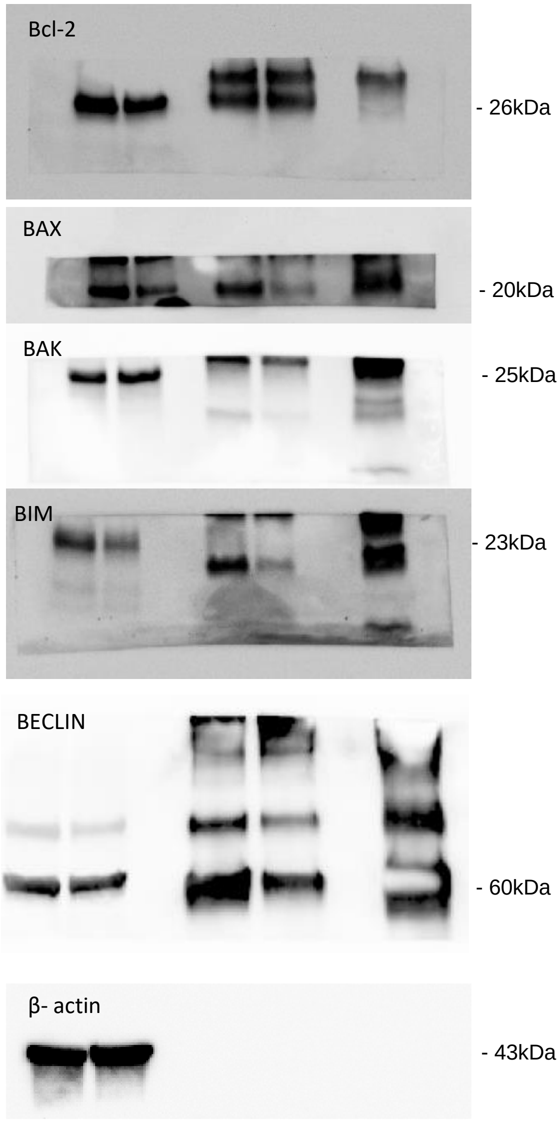
Bcl-2
- 26kDa
BAX
- 20kDa
BAK
- 25kDa
BIM
- 23kDa
BECLIN
- 60kDa
β- actin
- 43kDa

FIG2H

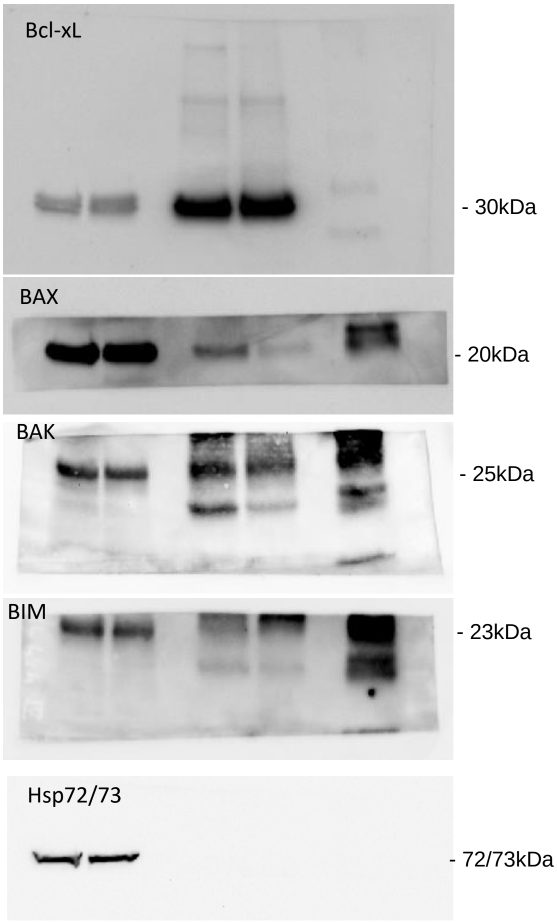

Bcl-xL
- 30kDa
BAX
- 20kDa
BAK
- 25kDa
BIM
- 23kDa
Hsp72/73
- 72/73kDa

FIG2I

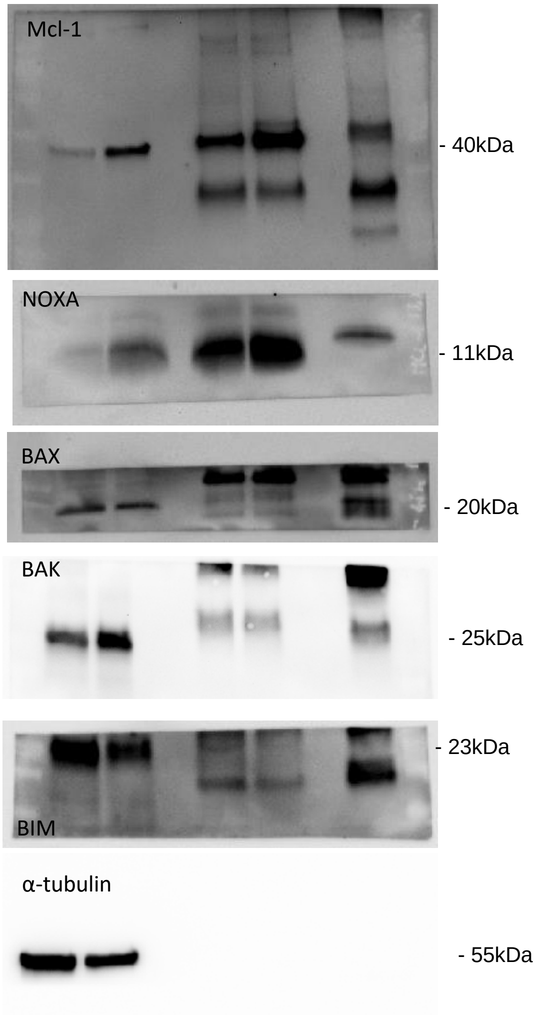

Mcl-1
- 40kDa
NOXA
- 11kDa
BAX
- 20kDa
BAK
- 25kDa
- 23kDa
BIM
α-tubulin
- 55kDa

SUPPLEMENTARY FIG 1D

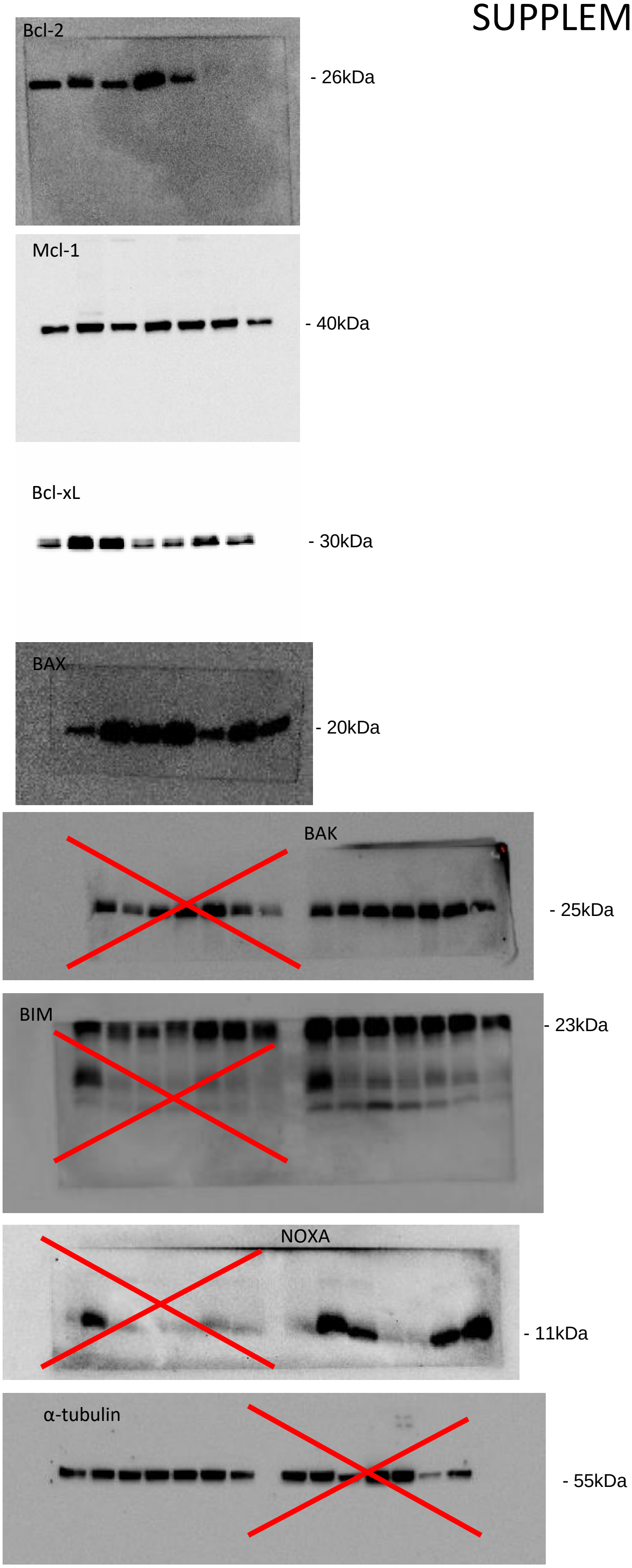

SUPPLEMENTARY FIG 1F

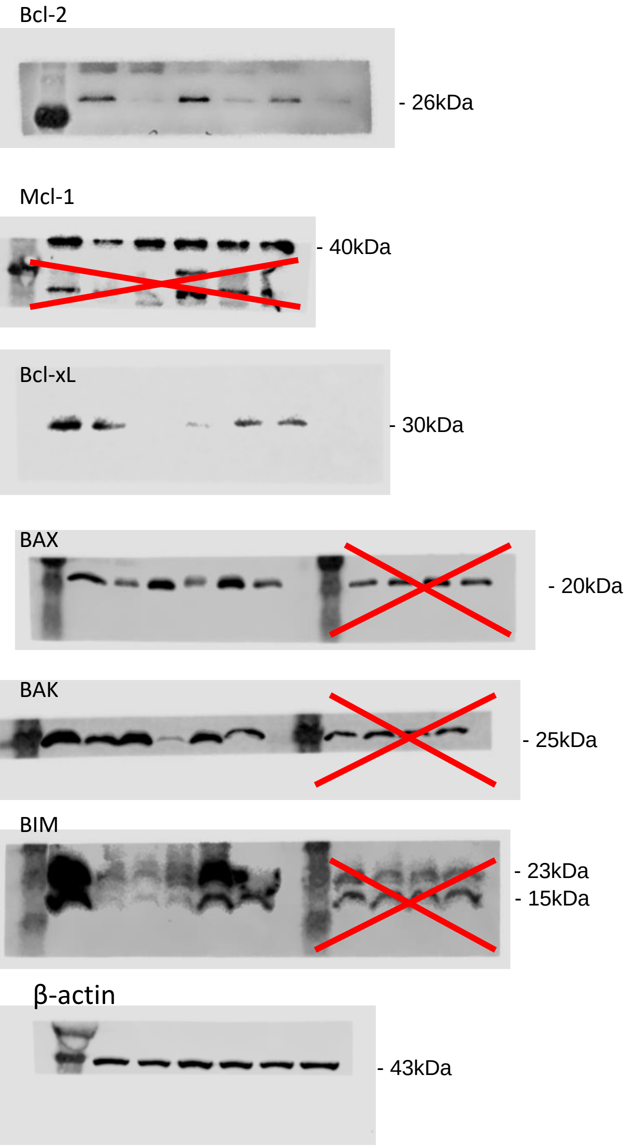

SUPPLEMENTARY FIGURE 2B

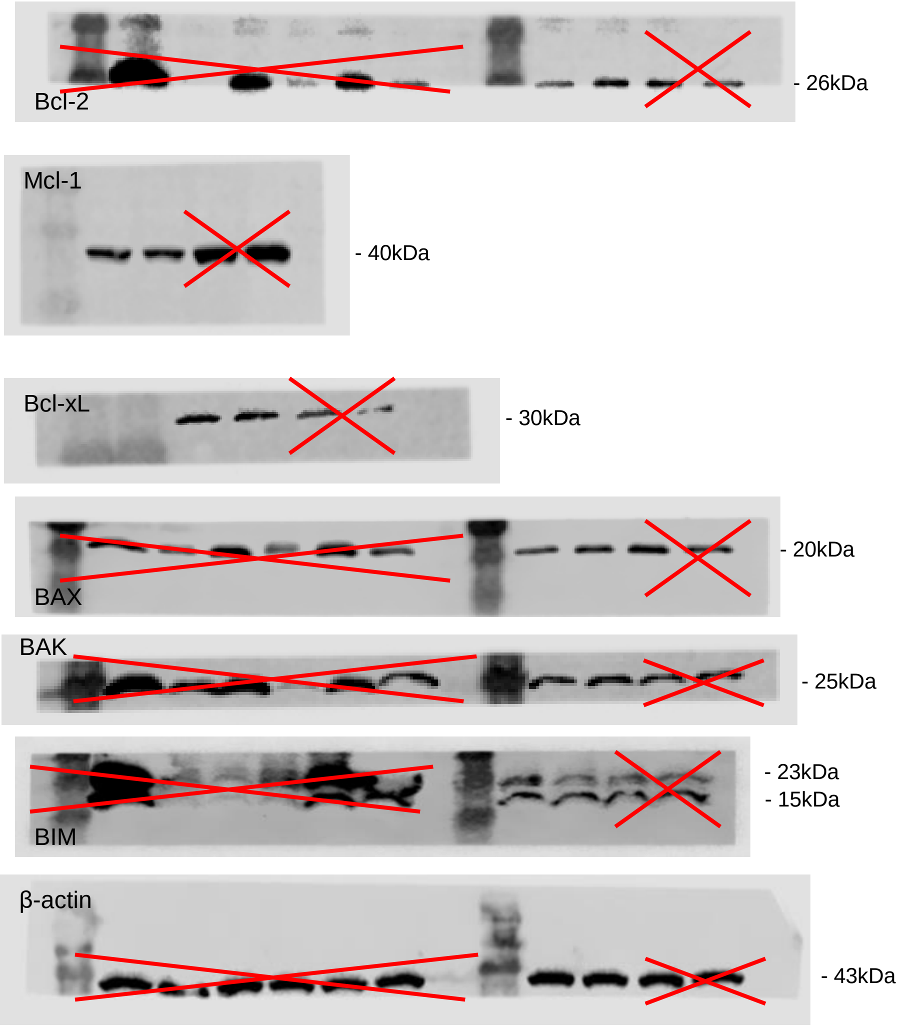

SUPPLEMENTARY FIGURE 3B

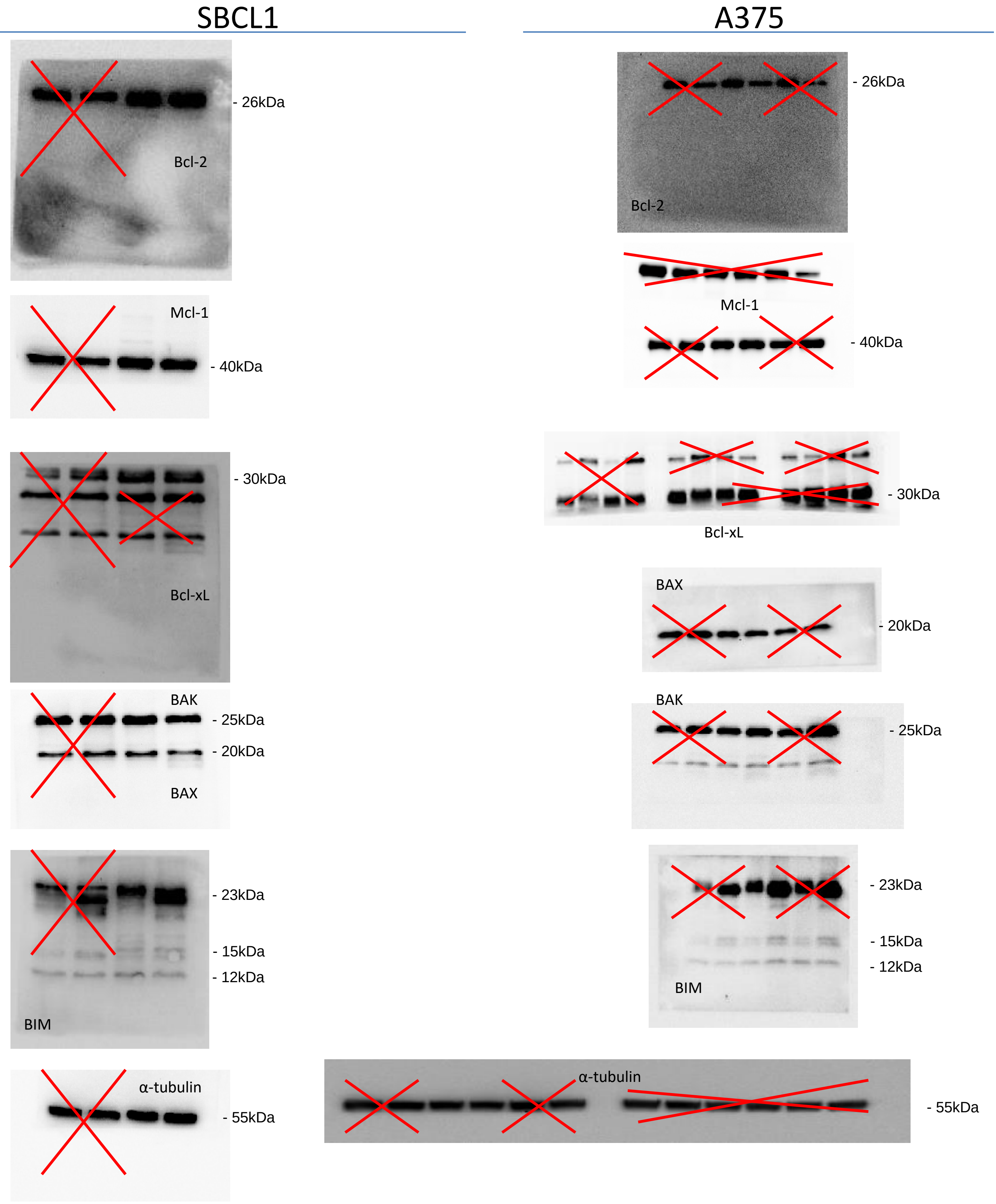

Supplement: Supplementary file 1 — Original Data File [file 41419_2023_5963_MOESM1_ESM.pdf]
