# Supplementary material for: Bcl-2 family inhibitors sensitize human cancer models to therapy
Source: Cell Death Dis. 2023 Jul 17;14(7):441. doi: 10.1038/s41419-023-05963-1 (PMC10352371; doi:10.1038/s41419-023-05963-1)
Supplement: Supplementary file 2 — Supplementary file [file 41419_2023_5963_MOESM2_ESM.pdf]

Supplementary Files

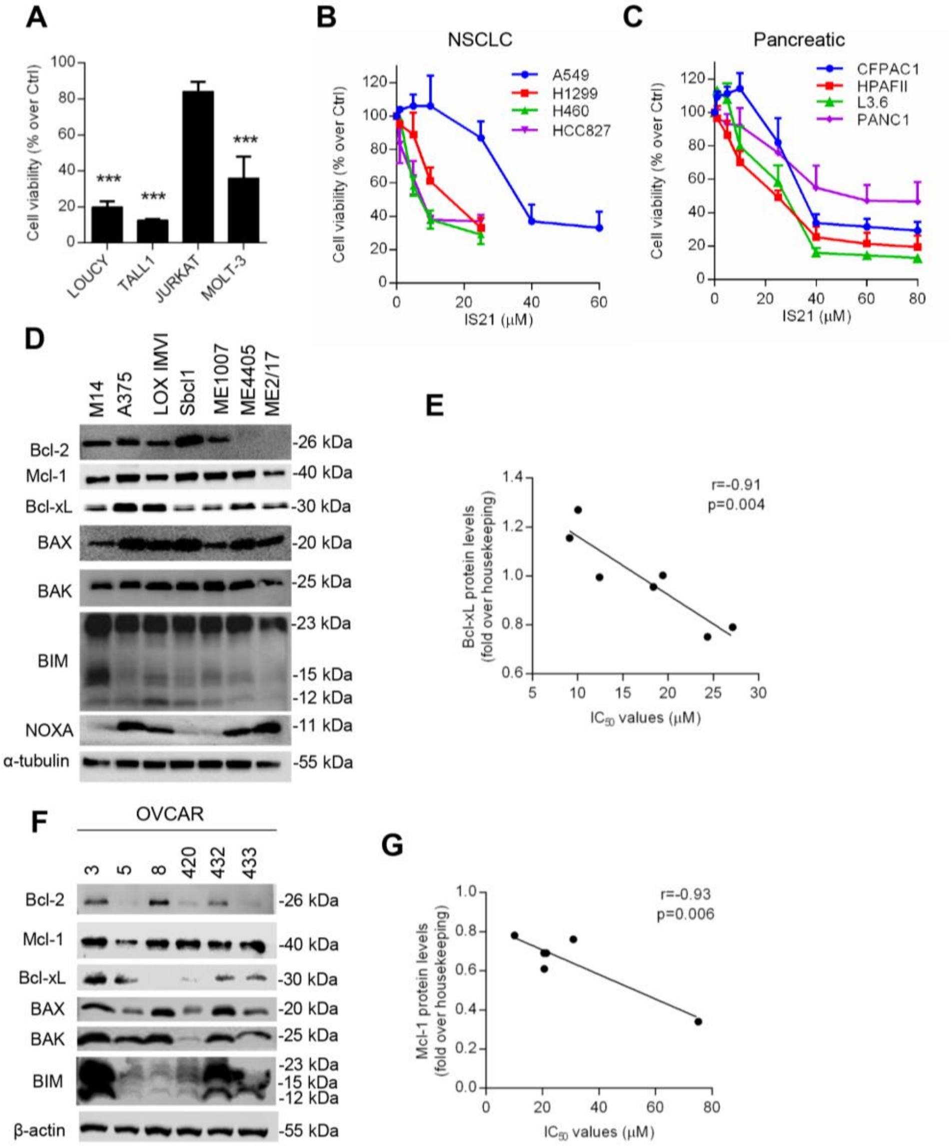

Supplementary Fig. 1. ***(A)*** *Analysis of cell viability of the indicated T-ALL cell lines treated with ABT-199 (1µM, 24h). p-values were calculated between control and treated cells, ***p ≤0.001.* ***(B,C)*** *Analysis of cell viability of* ***(B)*** *NSCLC and* ***(C)*** *pancreatic cancer cell lines treated with increasing concentrations of IS21 for 72h.* (A-C) *The results are reported as "viability of treated cells/viability of control cells (Ctrl)" × 100, and as mean ± SD of three independent experiments.* ***(D)*** *Western blot analysis of basal Bcl-2, Mcl-1, Bcl-xL, BAX, BAK, BIM and NOXA protein expression levels in the indicated melanoma cell lines.* ***(E)*** *Correlation analyses between* IC_{50} *IS21 values and the basal levels of Bcl-xL expression in melanoma cell lines.* ***(F)*** *Western blot analysis of basal Bcl-2, Mcl-1, Bcl-xL, BAX, BAK and BIM protein expression levels in the indicated ovarian cancer cell lines.* ***(G)*** *Correlation analyses between IC50 values of IS21 and the basal levels of Mcl-1 expression in ovarian cancer cell lines.* ***(D,F)*** *Reported Western blot images are*

representative of two independent experiments with similar results. β-actin and α-tubulin are shown as loading and transferring control, molecular weights are expressed in kilodalton (kDa).

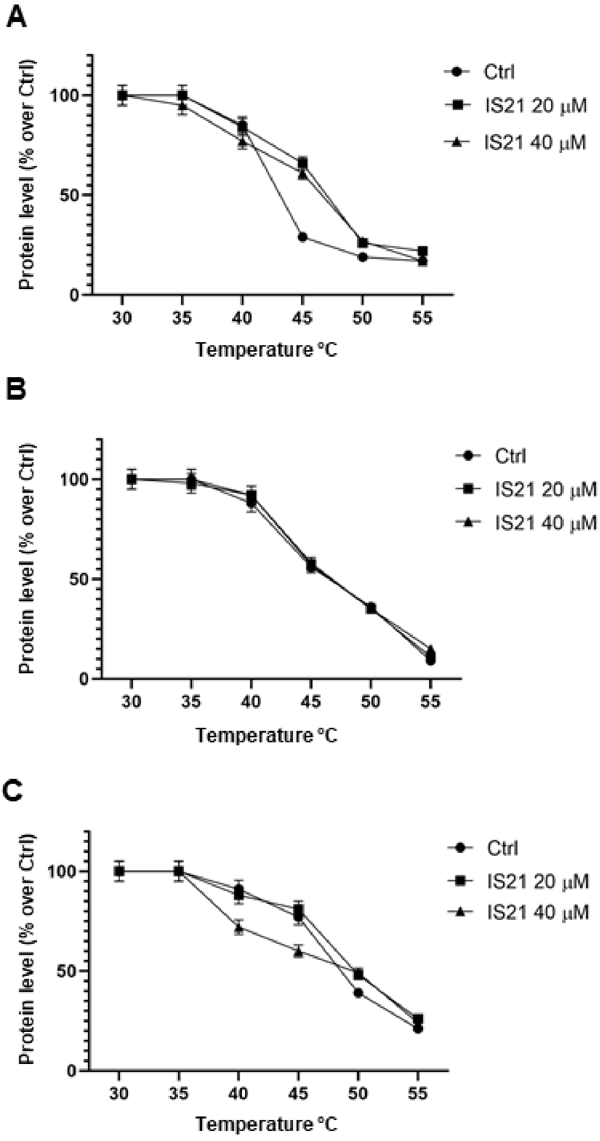

Supplementary Fig. 2. *Intracellular thermal denaturation curves obtained for (**A**) Bcl-2, (**B**) Bcl-xL and (**C**) Mcl-1 in A375 cells underwent a 4 h treatment with IS21 or untreated (Ctrl).*

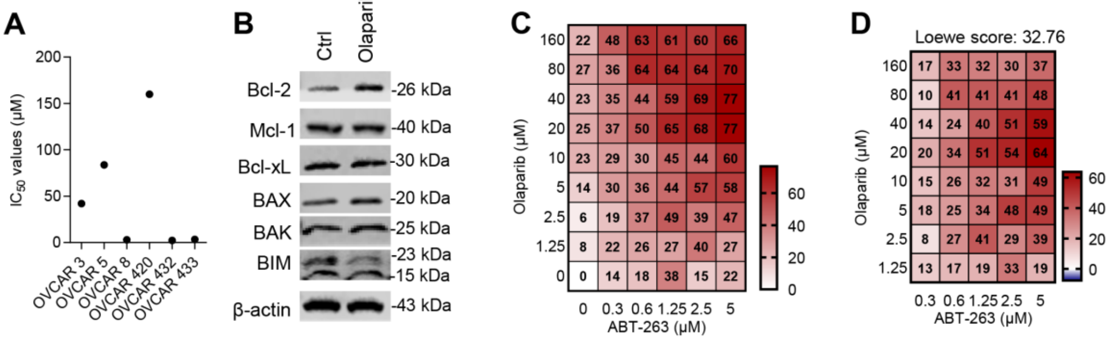

Supplementary Fig. 3. ***(A)*** *IC_{50} values of the indicated ovarian cancer cell lines treated with increasing concentration of olaparib (0.3-5µM,6 days).* ***(B)*** *Western blot analysis of Mcl-1, Bcl-xL, Bcl-2, BAX, BIM and BAK protein expression levels in OVCAR 5 ovarian cancer cell line treated with olaparib (5µM, 72h). Reported Western blot images are representative of two independent experiments with similar results. β-actin is shown as loading and transferring control, molecular weights are expressed in kilodalton (kDa).* ***(C,D)*** *Heat map graphs showing* ***(C)*** *the cell growth inhibitory effect, analyzed by MTS assay, and* ***(D)*** *Loewe scores of OVCAR 5 cells treated with the indicated concentrations of ABT-263 and olaparib alone or in combination for 6 days.* ***(C)*** *The results are reported as inhibition of treated cells/inhibition of control cells x 100 and represented the mean of three independent experiments.* ***(D)*** *The numbers inside the squares indicate Loewe scores in every experimental point.*

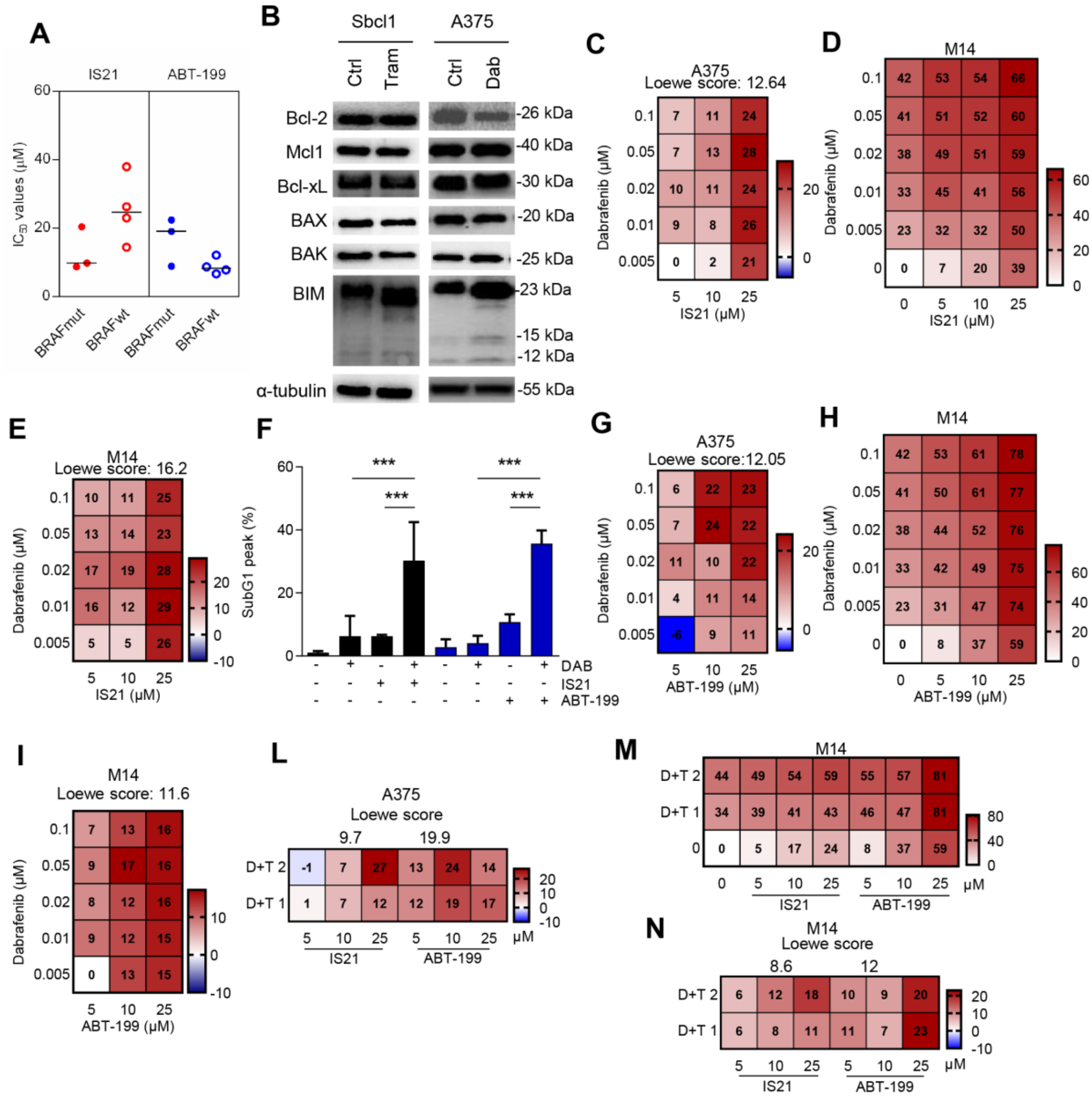

Supplementary Fig. 4. ***(A)*** *IC_{50} values of different melanoma cell lines (BRAFwt and BRAFmut) treated with IS21 or ABT-199 (from 5µM to 25µM for 72 h). The bar indicates the median of IC_{50} for each condition.* ***(B)*** *Western blot analysis of Mcl-1, Bcl-xL, Bcl-2, BIM, BAX and BAK protein expression levels in Sbcl1 and*

A375 cancer cell lines treated with trametinib (10nM) or with dabrafenib (0.1µM) for 48h, respectively. Reported Western blot images are representative of two independent experiments with similar results. α-tubulin is shown as loading and transferring control, molecular weights are expressed in kilodalton (kDa). ***(C)*** *Heat map graph showing Loewe scores of A375 cells treated for 48h with the indicated concentrations of dabrafenib (DAB) and IS21 alone or in combination. Heat map graphs showing (**D**) cell growth inhibitory effect and (**E**) Loewe scores of M14 cells treated for 48h with the indicated concentrations of dabrafenib and IS21 alone or in combination.* ***(F)*** *Cytofluorimetric quantification of subG1 peak of cell cycle distribution in M14 cells treated with IS21 (25µM) or ABT-199 (10µM) alone or in combination with Dabrafenib (0.1µM for IS21 combination and 0.05µM for ABT-199). Data represented the mean ± SD of three independent experiments. p-values were calculated between single treatments and combination ones, ***p<0.001. (**G**) Heat map graph showing Loewe scores of A375 cells treated for 48h with the indicated concentrations of dabrafenib and ABT-199 alone or in combination. Heat map graphs showing (**H**) cell growth inhibitory effect and (**I**) Loewe scores of M14 cells treated with the indicated concentrations of dabrafenib and ABT-199 alone or in combination. (**L**) Heat map graph showing Loewe scores of A375 cells treated with IS21 or ABT-199 alone or in combination with dabrafenib+trametinib (D+T 1, dabrafenib 0.01µM+trametinib 0.001µM; D+T 2, dabrafenib 0.05µM+trametinib 0.005µM) for 48h. Heat map graphs showing (**M**) cell growth inhibitory effect and (**N**) Loewe scores of M14 cells treated with IS21 or ABT-199 alone or in combination with dabrafenib+trametinib (D+T 1, dabrafenib 0.01µM+trametinib 0.001µM; D+T 2, dabrafenib 0.05µM+trametinib 0.005µM) for 48h.* ***(D,H,M)*** *The results are reported as inhibition of treated cells/inhibition of control cells x 100 and represented the mean of three independent experiments.* ***(C,E,G, I,L,M)*** *The numbers inside the squares indicate Loewe and inhibition values in every experimental point.*

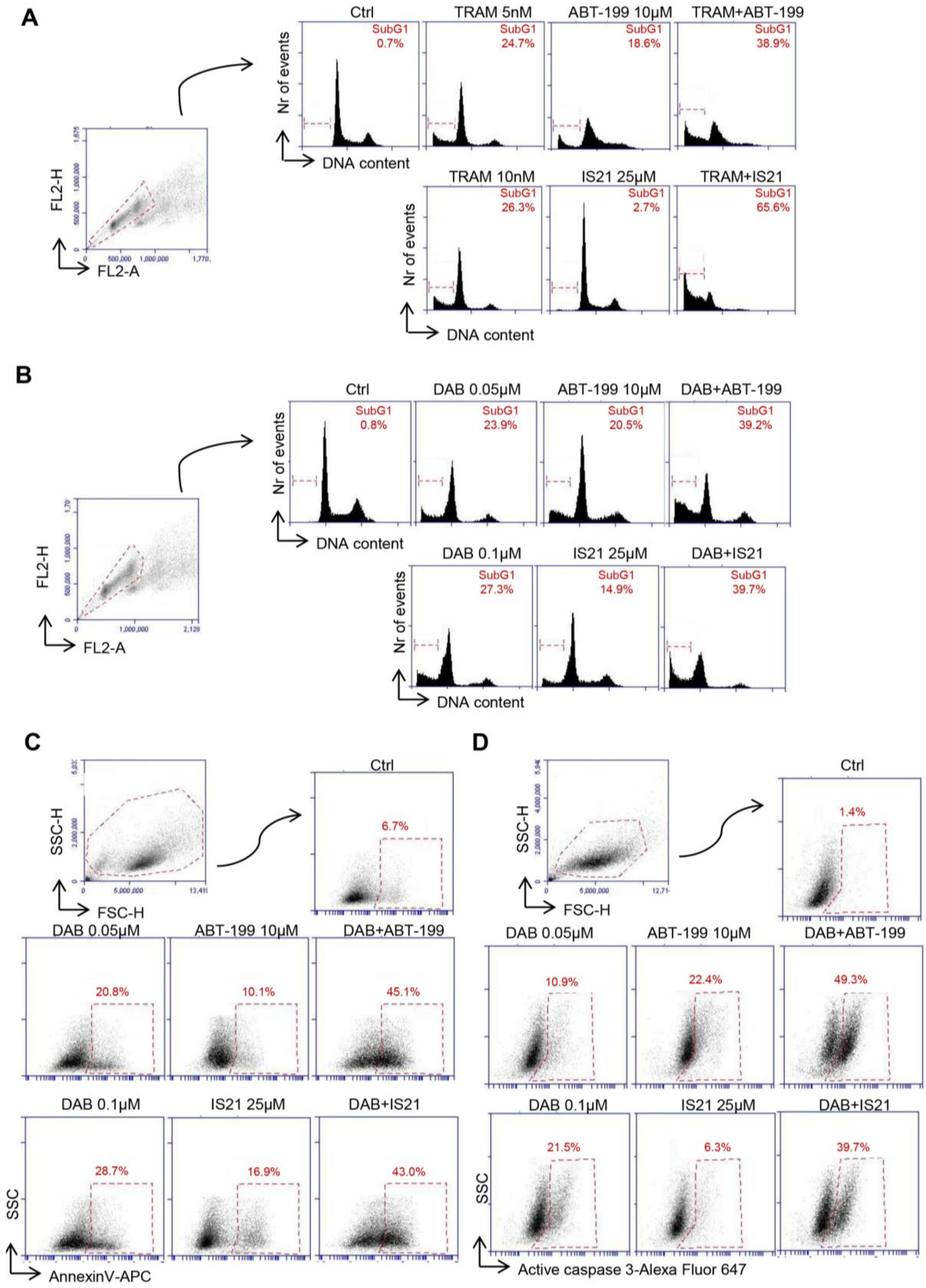

Supplementary Fig. 5. *Representative images of flow cytometric analysis of subG1 peak in (**A**) Sbcl1 cells after treatment with the indicated concentrations of trametinib (TRAM), IS21, ABT-199 alone or in combination (TRAM+IS21, TRAM+ABT-199) for 48h and (**B**) A375 cells treated with the indicated concentrations of dabrafenib (DAB)**,** IS21, ABT-199 alone or in combination (DAB+IS21, DAB+ABT-199)*

*for 48h. Representative images of (**C**) AnnexinV and (**D**) active caspase 3 staining of A375 treated as reported in (**B**).*

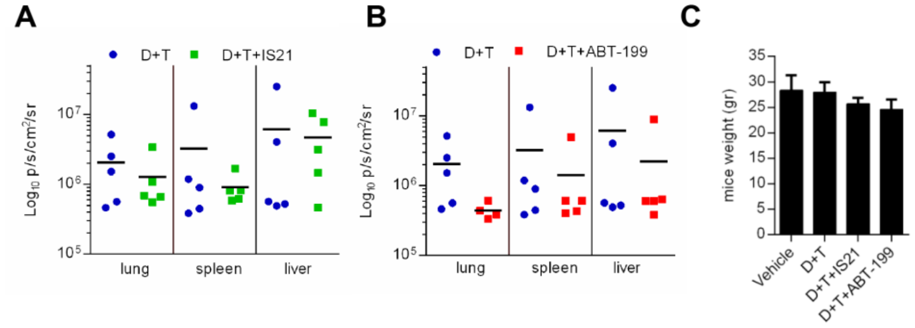

Supplementary Fig. 6. *Analysis of the metastatization measured by ex vivo imaging on lung, spleen and liver of mice treated for three weeks with dabrafenib+trametinib (D+T) and (**A**) D+T+IS21 or (**B**) D+T+ABT-199. (**C**) Analysis of mice weight after treatment reported in (A,B).*

Bcl Protein Type	pdb entry code	Docking Energy (kcal/mol)*		KD_1 (µM)
		Raw	Minimized	
Bcl-2	2VM6	-7.5	-7.72	0.32
Bcl-xL	3FDL	-8	-8.1	0.42
Mcl-1	2PQK	-6.7	-7.39	3.9

Supplementary Table 1. *Predicted docked energy by the VINA scoring function for IS21 as docked into Bcl-2, Bcl-xL and Mcl-1 proteins extracted from complexes BH3 alpha-helix. *Data are the average values from two independent docking runs from two different randomized starting conformations.*
